# Tissue-resolved transcriptomic and proteomic profiling reveals organ-specific molecular signatures associated with inter-animal variation in glycemic response to metformin in obese diabetic Mongolian gerbils

**DOI:** 10.3389/fendo.2026.1954533

**Published:** 2026-10-23

**Authors:** Zheng Gao, Hongjuan Fang, Feiyang Li, Mo Wang, Zishan Zhang, Xueyun Huo, Xiaoyan Du

**Affiliations:** 1 Clinical Medicine Laboratory, Department of Medical Genetics and Developmental Biology, School of Basic Medical Sciences, Capital Medical University, Beijing, China; 2 Department of Endocrinology, Aviation General Hospital, Beijing, China

**Keywords:** metformin, Mongolian gerbils, multi-omics, tissue-resolved profiling, type 2 diabetes mellitus

## Abstract

**Background:**

Type 2 diabetes mellitus (T2DM) affects hundreds of millions of people worldwide. Metformin remains first-line therapy for obese T2DM; yet, 30–40% of patients fail to achieve adequate glycemic control with monotherapy, the magnitude of HbA1c reduction varies two- to threefold between individuals, and the multi-organ coordination underlying this inter-animal variation in glycemic response remains unclear.

**Methods:**

Obese diabetic Mongolian gerbils were administered metformin (100 mg/kg/day, oral gavage; n = 4) or vehicle (n = 3) for 5 weeks. We performed matched transcriptomic (RNA-seq) and data-independent acquisition proteomic profiling of liver, skeletal muscle, and inguinal white adipose tissue. Cross-omics analyses were anchored to glycemic parameters, and key hepatic findings were validated by quantitative real-time polymerase chain reaction (qRT-PCR). Gut microbiota were profiled via metagenomic sequencing.

**Results:**

Metformin lowered 2-hour post-load glucose (2hPG) and ameliorated hepatic steatosis. In the liver, integrated transcriptomic–proteomic analysis revealed concordant downregulation of mitochondrial substrate carriers (Slc25a47, Slc25a29, Slc25a30) and lipid-handling factors (e.g. Apoa5, Apoc2), correlating with systemic glycemic burden among treated animals. In skeletal muscle, pathway-level integration supported the activation of metabolic remodeling programs, including PPAR signaling, that were tracked with the degree of glycemic improvement among treated animals. In iWAT, transcriptomic enrichment of immune and chemokine pathways, supported by proteomic trends, suggested that, among treated animals, higher residual inflammatory activity was associated with a weaker glycemic response. Gut metagenomic profiling revealed no detectable alterations in microbial diversity or community structure.

**Conclusion:**

Hepatic mitochondrial-lipid remodeling and muscle metabolic activation aligned with glycemic improvement, while adipose tissue immune signatures may contribute to inter-animal variation in glycemic response, providing a multi-organ, data-supported framework for metformin’s systemic pharmacology.

## Introduction

Type 2 diabetes mellitus (T2DM) affects 589 million adults worldwide (2024) and is projected to rise to 853 million by 2050 (1). About four in five patients also carry obesity, which fuels insulin resistance and metabolic dysfunction (2). Metformin remains the first-line drug for obese T2DM and carries the strongest ADA recommendation; yet, its benefits are uneven (3). Some 30–40% of patients do not achieve adequate glycemic control on metformin alone, and HbA1c reductions can differ two- to threefold between individuals (4–6). The tissue-level causes of this split remain poorly understood.

Most studies have examined the liver, skeletal muscle, and adipose tissue in isolation. The liver suppresses gluconeogenesis and reshapes lipid metabolism through AMPK-dependent and AMPK-independent pathways. Muscle governs whole-body glucose disposal by taking up and oxidizing glucose. Adipose tissue functions as an immunometabolic hub where adipokines and infiltrating immune cells modulate insulin sensitivity. Single-organ work has identified important targets such as AMPK, but it cannot capture how metformin redistributes metabolic and inflammatory signals across organs. Mitochondrial function, bile acid signaling, and inflammatory pathways all contribute, and their relative importance differs by tissue (6, 7). A tissue-resolved multi-omics approach is therefore needed.

We profiled liver, skeletal muscle, and inguinal white adipose tissue (iWAT) with matched RNA-seq and DIA-MS proteomics, plus gut metagenomics, in spontaneously obese diabetic Mongolian gerbils. This model develops obesity, insulin resistance, hyperglycemia, and hepatic steatosis without dietary manipulation, so it offers a genetically coherent platform for studying drug response (8, 9). We present a tissue-resolved transcriptomic and proteomic profiling framework that maps metformin’s organ-specific molecular programs and links them to individual glycemic response. This methodology—anchoring matched transcriptomic-proteomic signatures to phenotype at the individual-animal level—provides a scalable strategy for dissecting inter-animal variation in glycemic response in complex metabolic diseases.

## Materials and methods

### Animal model and treatment

The Animal Ethics and Experimental Safety Inspection Committee of Capital Medical University approved all animal experiments (Approval No. AEEI-2021-052).

We used male gerbils (8–10 months; body weight at enrollment 88–122 g) from our inbred obese diabetic line as the model (7, 10, 11). Animals were maintained under specific pathogen–free (SPF) conditions (22 ± 2 °C, 50 ± 10% relative humidity, 12-h light/dark cycle) with free access to sterile water and standard maintenance chow. Diabetes was defined as fasting plasma glucose (FPG) > 5.2 mmol/L and 2-h post-load glucose (2hPG) > 6.8 mmol/L. Eligible animals were then assigned to metformin (100 mg/kg/day, oral gavage) or vehicle for 35 days (n = 4 treatment, n = 3 control) by simple randomization (drawing lots), without stratification or blocking. No statistically significant baseline differences in body weight, FPG, or 2hPG were detected between the two groups (all P > 0.19); per-animal baseline values and serum biochemistry at baseline and week 5 are provided in **Supplementary Table S1**, where baseline serum TC and LDL-C were higher in the metformin group by chance of simple randomization in this small cohort (P = 0.037 and 0.024) and are reported transparently. To ensure phenotypic homogeneity of the molecular baseline, all omics differential analyses were restricted to animals with the spontaneous obese diabetic (OD) phenotype.

Metformin was prepared from commercial Metformin Hydrochloride Tablets (Glucophage, SinoUS Shanghai Squibb Pharmaceutical Co., Ltd.), dissolved in double-distilled water, and administered via oral gavage at 100 mg/kg/day (dose expressed as metformin hydrochloride; 10 mL/kg body weight) for 35 consecutive days. This dose was chosen because 100 mg/kg/day is a widely used and effective oral dose in diabetic or obese rodent models (spontaneously diabetic KKAy mice for 10 weeks (12); streptozotocin-induced diabetic mice for 28 days (13); high-fat-diet-induced obese C57BL/6J mice for 6 weeks (14)) and corresponds, by body-surface-area conversion (15), to a human-equivalent dose of ≈ 13.5 mg/kg/day (≈ 810 mg/day for a 60-kg adult), at the lower end of the clinical range (500–2, 550 mg/day); the higher doses commonly used in rodent studies (250–500 mg/kg) were avoided to prevent appetite- and weight-related confounding of the weight-independent efficacy readouts. Because the drug was prepared from commercial tablets, trace excipients (e.g., starch, povidone, and magnesium stearate) were present in the dosing solution, whereas the vehicle was double-distilled water only; this is acknowledged as a limitation. The control group received an equal volume of double-distilled water on the same administration schedule. Solutions were prepared and oral gavage was performed by the same investigator; outcome assessment (blood glucose measurement and histology scoring) was performed blinded to group allocation; omics data were analyzed under coded group labels and were unblinded only after analyses were complete.

### Metabolic and histological phenotyping

We monitored blood glucose weekly and, at endpoint, performed an oral glucose tolerance test (OGTT; 2 g/kg) after a 16-h fast. Treatment response was quantified as ΔFPG and Δ2hPG (week 5 minus baseline). Terminal serum lipids (TC, TG, HDL-C, LDL-C) and liver enzymes (ALT, AST) were measured on a BS-240VET analyzer.

At endpoint, gerbils were anesthetized with sodium pentobarbital (40 mg/kg, intraperitoneal). Under deep anesthesia, blood was collected from the retro-orbital venous plexus, and animals were euthanized by exsanguination; death was confirmed by cessation of heartbeat and respiration before tissue collection. Following euthanasia, liver, skeletal muscle, and inguinal white adipose tissue (iWAT) were rapidly excised. Portions for omics assays were snap-frozen in liquid nitrogen and stored at −80 °C. Tissues for histology were fixed in 4% paraformaldehyde at 4 °C for 24 h, dehydrated through a graded ethanol series, embedded in paraffin, and sectioned at 5 μm thickness.

### RNA sequencing and transcriptomic analysis

RNA extraction and quality assessment were performed by Novogene Technology Co., Ltd. (Beijing, China) as part of the standardized sequencing workflow; total RNA was extracted from the snap-frozen liver, skeletal muscle, and iWAT samples described above with TRIzol reagent (Invitrogen, USA), the vendor’s standard extraction method for animal tissues. RNA concentration and purity were measured with a NanoDrop spectrophotometer (A260/A280), and RNA integrity (RIN and 28S/18S) was determined on an Agilent 2100 Bioanalyzer (Agilent Technologies, USA); only samples passing the vendor acceptance criteria proceeded to library construction. RIN values and 28S/18S ratios for all samples are provided in **Supplementary Table S2**; all sequenced samples passed vendor quality control (liver RIN 8.5–9.0; skeletal muscle RIN 6.6–8.5; iWAT RIN 7.1–8.2), and no statistically significant differences in RIN were detected between treatment groups in any tissue (all P > 0.10).

Transcriptome sequencing and initial data processing were conducted by Novogene Technology Co., Ltd. (Beijing, China) following a standard commercial protocol. Briefly, poly(A)+ mRNA was enriched with oligo(dT) magnetic beads for cDNA library construction. Libraries were sequenced on an Illumina NovaSeq 6000 platform (paired-end 150 bp) using the Mongolian gerbil reference genome assembly MunDraft-v1.0 (Ensembl Release 111).

Raw reads were filtered to remove low-quality sequences, adapters, and contaminants. Clean reads were aligned to the MunDraft-v1.0 reference genome using HISAT2 (v2.0.5) (16), and gene-level read counts were generated with featureCounts (v1.5.0-p3) (17). Sequencing quality was high across all samples (Q30 ≥ 94.8%; mapping rates 87–94%; **Supplementary Table S2**). Differential expression between the metformin-treated (OD + Met) and vehicle control (OD) groups was analyzed with the DESeq2 R package (18). P values were adjusted using the Benjamini–Hochberg procedure (19), and genes with an adjusted P-value < 0.05 were considered differentially expressed. For principal component analysis (PCA) and correlation analyses, variance-stabilized (rlog-transformed) read counts were employed (18).

### qRT-PCR validation

Key findings were validated by qRT-PCR and Western blot. For qRT-PCR, total RNA was reverse-transcribed and amplified with SYBR Green chemistry on a Bio-Rad C1000 system, with expression normalized to Gapdh. All primers were designed with NCBI Primer-BLAST, synthesized by Tianyi Huiyuan (Beijing, China), and used at a uniform annealing temperature of 60 °C; amplicon lengths and Tm values are listed in **Table 1**, and full primer details including GC content and RefSeq templates are provided in **Supplementary Table S5**. For Western blot, proteins (30 μg) were separated by SDS-PAGE, transferred to PVDF, and probed with antibodies against Slc25a47, Prkag2, and Sirt3 (Proteintech, 1:1000), detecting signals by ECL, quantifying them in ImageJ, and normalizing them to β-actin or GAPDH.

**Table 1:** PCR primer sequences.

Gene	Forward	Reverse	Amplicon (bp)	Tm (°C, F/R)
Slc25a47	CGCCTCTGCTCACAGGTATTT	ATGGCCCCAGCAACAAAATC	101	60.4/59.4
Slc25a29	CTCGTGGGACACCCTTTTGA	GTCTCACGCAGAAGTGTGGA	428	59.9/60.0
Slc25a30	TGTGTGTGGAATCCTGTCCG	TCTGGGCTTGCATTCGGATT	83	60.0/60.0
Apoc2	AGAGGAGCTGCCTGATATGG	TCCCGATTCCTGAACGTAACA	112	58.7/59.1
Apoa5	AGAACTGAGCAGGGCGCAT	AGAAACTCTTCCGTGCCTGAG	102	62.0/60.0
Lepr	CAGACCCTGAGAGGGGTTCT	CGCACCTGCTGACAGAACTA	192	60.3/60.0
Myef2	GCATTGGAATGGGACTTGGC	ATTCCTCCAAAACCCGGACC	124	59.8/60.0
Apob	GCTGAGTGTCTACTTCCACCC	TCACACGTTCCAGGAAGGATA	148	60.1/58.5
Ccl19	CCATCACTCCCCTGAACACC	GCTAAAGGCCAGTAGGACGG	147	60.0/60.2
Ccr7	TACAACGGAGTGGTTCTGGC	AGGCCAGGCTGTAGGTGATA	112	60.0/60.0
Cd40	CTCTGTGGTTTCCAGCTCCG	GTTGGTTTCTTGGCCACCTTT	122	60.7/59.5
Cd40lg	CAGCCAAACTTCTCCCCGAT	TAAAGCGCCGACCCAATCAT	111	60.04/60.11
β-actin	AGTCGGTTGGAGCAAGCATC	GGTGAGGGGCTTCCTGTAGT	119	60.7/60.9
GAPDH	GACACGAGCAAGAGAGAGGC	TCAAGAGTAGGGCTCCCTGTG	131	60.5/60.9

All primers were designed with NCBI Primer-BLAST on Meriones unguiculatus RefSeq templates, synthesized by Tianyi Huiyuan (Beijing, China), and used at a uniform annealing temperature of 60 °C. Cxcl13 and Cxcr5 primer pairs were removed because these two genes were not among the qRT-PCR-validated targets.

### Proteomics sample preparation, DIA-MS acquisition, and protein quantification

Frozen liver, skeletal muscle, and iWAT samples were pulverized in liquid nitrogen and lysed in buffer containing 100 mM NH4HCO3 (pH 8), 6 M urea, and 0.2% SDS, followed by ultrasonication on ice for 5 min. After centrifuged at 12, 000 × g for 15 min at 4 °C, the supernatant was collected, and protein concentration was determined by Bradford assay. Proteins were reduced with 2 mM dithiothreitol (56 °C, 1 h), alkylated with iodoacetamide (room temperature, 1 h, dark), and precipitated with four volumes of cold acetone at −20 °C for 4 h. The pellet was washed twice with cold acetone, redissolved in 0.1 M triethylammonium bicarbonate (TEAB, pH 8.5) with 6 M urea, and digested with trypsin (1:100 w/w) at 37 °C overnight. Peptides were acidified with 1% formic acid, desalted on a C18 column, and lyophilized.

Peptide separation and data-independent acquisition mass spectrometry (DIA-MS) were performed by Novogene Co., Ltd. (Beijing, China) using a standardized commercial pipeline for mammalian tissue proteomics. Lyophilized peptides were separated on a Vanquish™ Neo UHPLC system (Thermo Fisher Scientific) equipped with a C18 trap column (5 mm×300 μm, 5 μm) and a PepMap™ Neo UHPLC (150 μm×15 cm, 2 μm) with a gradient of 0.1% formic acid in water (A) and 0.1% formic acid in 80% acetonitrile (B) at 300 nL/min. MS was performed on an Orbitrap Astral mass spectrometer (Thermo Fisher Scientific) in DIA mode: full MS scans were acquired at 120, 000 resolution (m/z 400–1000), followed by DIA MS/MS scans at 30, 000 resolution with 25% normalized collision energy.

Raw DIA data were processed with DIA-NN (v1.8) (20) against the UniProt Mongolian gerbil proteome database (Taxonomy ID: 10047). Protein identifications were filtered at a 1% false discovery rate (FDR) at both the precursor and protein-group levels (Global.Q.Value < 0.01 and PG.Q.Value < 0.01). In the revised pipeline, proteins were first restricted to those detected in ≥50% of samples within at least one group, and remaining missing values were imputed by group-aware k-nearest neighbors (k = 2) (21); results were additionally verified against the original global KNN (k = 10) pipeline and a no-imputation pipeline, with candidate-gene membership reported under all three strategies in **Supplementary Table S4**. Differential abundance between the metformin-treated and vehicle control groups was assessed by a two-sample t-test; proteins with a nominal P < 0.05 and a fold change (FC) > 1.2 or < 0.83 were considered differentially abundant. Because of the small sample size, protein-level P values were not subjected to additional multiple-testing correction; these protein changes are exploratory and need separate validation. We acknowledge the resulting threshold asymmetry between the FDR-controlled RNA layer and the nominal-P protein layer; candidate-gene membership under protein-layer Benjamini–Hochberg correction and a stricter criterion (nominal P < 0.01 with |log2FC| ≥ 1) is reported in **Supplementary Table S4**.

### Gut metagenomic profiling

Fecal DNA was extracted with a commercial kit. Libraries were prepared by random fragmentation, end repair, A-tailing, and adapter ligation, and then sequenced on an Illumina NovaSeq platform. After quality filtering, reads were classified via Kraken2 against the NCBI NR database. Alpha diversity (ACE, Chao1, Shannon, Simpson) and beta diversity (PCA, NMDS on Bray-Curtis dissimilarity) were computed in R. Group differences were tested using unpaired t-tests for alpha diversity and PERMANOVA with 999 permutations for beta diversity. LEfSe was used to identify differentially abundant taxa (LDA > 2.0, P < 0.05, applied as an exploratory threshold).

### Cross-omics integration and efficacy-linked module scoring

Transcripts and proteins were matched by gene symbol. Concordance was visualized in nine-quadrant plots of RNA-seq log2 fold change versus proteomic log2 fold change. “Both-significant” gene products were defined as those significant at both omics layers (RNA FDR < 0.05 and protein nominal P < 0.05). A stringent subset — hereafter referred to as “exploratory cross-layer candidate genes” — was further defined by requiring |log2FC| ≥ 0.585 (≥1.5-fold) in both layers with concordant directionality (sign (RNA log2FC) = sign(protein log2FC)). This candidate definition was applied uniformly across liver, skeletal muscle, and iWAT. At the pathway level, concordant KEGG pathways were those significant in RNA-seq (FDR < 0.05) and showing enrichment in the same direction at the proteomic layer (nominal P-value reported as supporting trend). For each concordant pathway, per-sample activity scores were computed separately for RNA and protein data. RNA scores were derived from the sample-level FPKM matrix (log_2_(FPKM + 1)-transformed). Features were z-scored across samples within each layer and tissue, and pathway activity was summarized as the mean z-score of member genes/proteins. An integrated module score was obtained by averaging RNA- and protein-layer pathway scores.

### Data integration and statistical analysis

Differential expression for RNA-seq was determined with DESeq2 (adjusted P < 0.05). For proteomics, two-sample t-tests (nominal P < 0.05, |FC| > 1.2) were used; remaining missing values were imputed by group-aware k-nearest neighbors (k = 2), as detailed in the proteomics section above. KEGG enrichment was performed with the clusterProfiler R package (hypergeometric test with Benjamini–Hochberg adjustment), and pathway maps were rendered with pathview. Because the Mongolian gerbil is itself a KEGG-annotated organism (organism code “mun”), no cross-species orthologous projection was required: gerbil gene models from the MunDraft-v1.0 assembly (Ensembl release 111; 28, 930 genes, including 21, 116 protein-coding) were annotated in the vendor pipeline against the species-specific KEGG GENES entries for M. unguiculatus via NCBI GeneID (ENTREZ) identifiers, and all annotated pathways therefore carry mun-prefixed identifiers (e.g., mun04010). KEGG pathway annotation covered 9, 386 genes (32.4%), GO annotation covered 12, 180 genes (42.1%), and UniProt-based homologous naming was achieved for 22, 333 genes (77.2%); the 19, 544 genes without KEGG pathway annotation (67.6%) were excluded from pathway-level analyses (KEGG database Release 109.0, January 2024, the release current at the time of analysis in March 2024; clusterProfiler v4.10, Bioconductor 3.18). Candidate-gene concordance required consistent directionality and |log_2_FC| ≥ 0.585 in both layers (RNA FDR < 0.05, protein P < 0.05). Pathway activity per sample was calculated as the mean z-score of member genes or proteins, and correlated module scores with ΔFPG/Δ2hPG using Spearman’s rank test, and present data as mean ± SEM. Between-group comparisons used the Student’s t-test or the Mann–Whitney U test after Shapiro–Wilk normality testing. Two analysis layers were distinguished throughout: treatment-associated changes, identified from the metformin-versus-vehicle comparison within OD animals, and response-associated variation, assessed as correlations with ΔFPG and Δ2hPG among the four metformin-treated animals; the latter are correlative and were not interpreted as evidence of mediation. No prespecified responder/non-responder threshold was applied; ΔFPG and Δ2hPG were analysed as continuous traits in all association analyses. Analyses were performed in R 4.4.2 and GraphPad Prism 10.1.2.

## Results

### Metformin ameliorates hyperglycemia and mitigates pancreatic-hepatic pathology in obese diabetic Mongolian gerbils

Metformin treatment significantly lowered 2hPG at week 5 (5.03 ± 0.28 vs 6.90 ± 0.36 mmol/L, Welch’s t-test P = 0.0138, Cohen’s d = −3.23; **Figure 1A**), and FPG at week 5 showed a non-significant reduction (4.75 ± 0.42 vs 6.03 ± 0.27 mmol/L, P = 0.0518, d = −1.80; **Figure 1A**) (weekly Welch’s t-tests, n = 4 treated vs n = 3 control). No statistically significant between-group differences in changes from baseline (ΔFPG, Δ2hPG) were detected (P = 0.53 and P = 0.52), and body-weight change was comparable between groups (−13.5 ± 3.5 vs −14.3 ± 4.6 g, P = 0.89), indicating that glycemic improvement was not attributable to weight loss. Individual-animal trajectories, per-week exact statistics, and body-weight changes are provided in **Supplementary Figure S2**.

**Figure 1:**
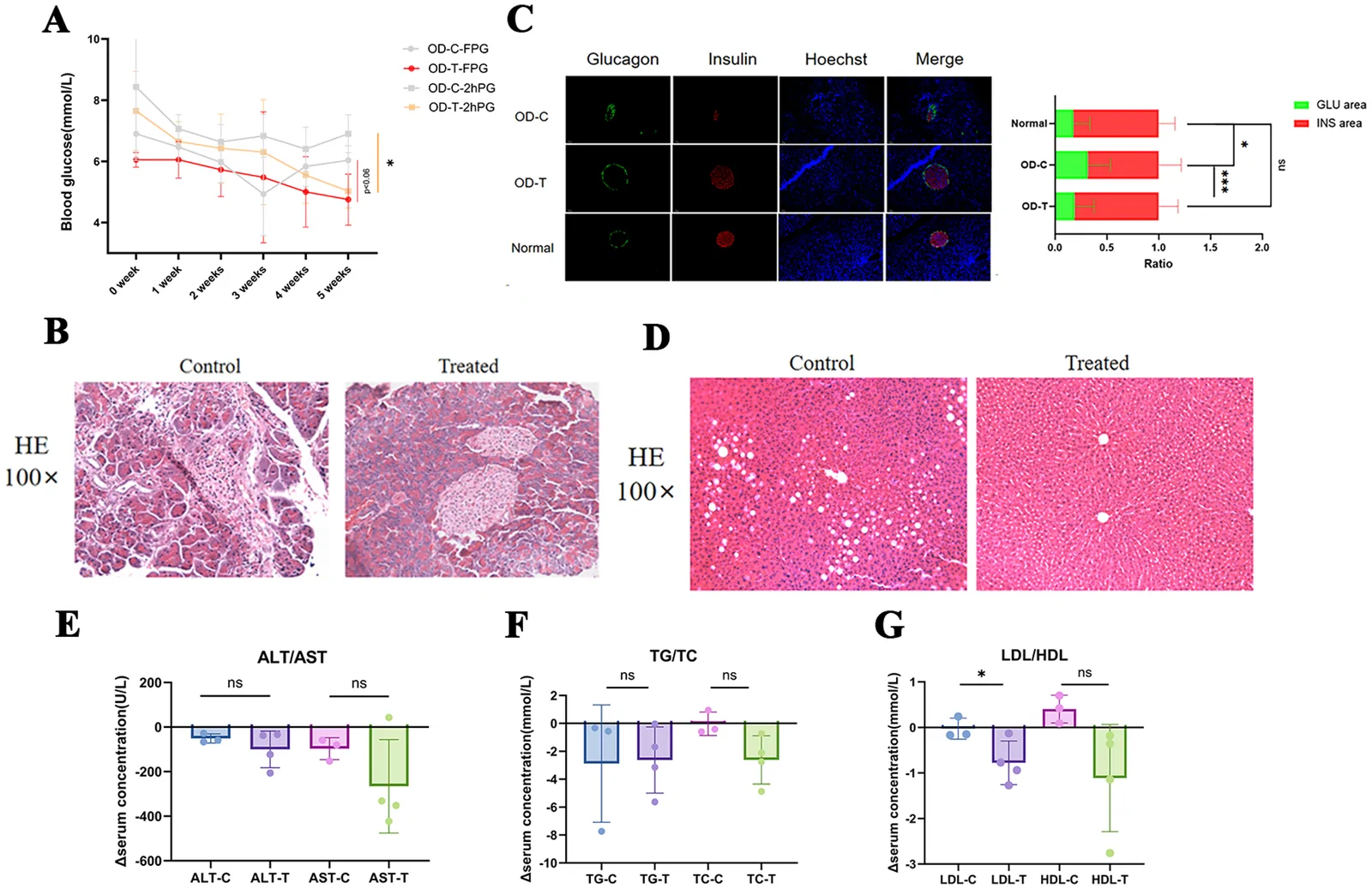
Metformin alleviates the glycemic burden and reverses pancreas–liver pathology in obese diabetic Mongolian gerbils. **(A)** Longitudinal trajectories of FPG and 2hPG over the 35-day intervention period. **(B, C)** Pancreatic islet integrity by H&E **(B)** and insulin immunofluorescence **(C)**, showing restored architecture and β-cell preservation in treated animals; **(D)** liver H&E staining showing reduced steatosis and lipid droplets after metformin; **(E–G)** changes from baseline (week 5 − week 0, Δ) in serum liver enzymes (ALT, AST) and lipid parameters (TG, TC, LDL-C, HDL-C), indicating improved lipid homeostasis. Data are presented as mean ± SEM (n = 4 treated and n = 3 control for glycemic and body-weight measures; n = 3–4 per group for serum and histological measures). Weekly glucose comparisons used Welch’s t-test. *P < 0.05, **P < 0.01, ***P < 0.001 vs. control; ns, not significant (Welch's t-test).

Histopathological analysis revealed that metformin partially restored disrupted pancreatic islet architecture observed in controls (**Figure 1B**). Immunofluorescence staining indicated an expanded insulin-positive area within islets after treatment (**Figure 1C**). In the liver, metformin reduced lipid droplet accumulation and improved hepatic morphology compared to controls (**Figure 1D**); quantitative analysis confirmed a significant reduction in hepatic lipid-droplet area (1.20 ± 0.26% vs 5.44 ± 0.91% of sectional area, Welch’s t-test P = 0.0346, Cohen’s d = 3.95, a 77.9% reduction). Concomitantly, at week 5 no statistically significant between-group differences in serum TC, LDL-C, or ALT were detected (TC 2.96 ± 0.08 vs 2.37 ± 0.27 mmol/L, P = 0.147; LDL-C 0.63 ± 0.01 vs 0.45 ± 0.12 mmol/L, P = 0.264; ALT 90.6 ± 1.6 vs 93.2 ± 3.2 U/L, P = 0.518; **Figures 1E–G**), whereas the within-group reduction from baseline was larger under metformin for LDL-C (Δ = −0.78 vs −0.03 mmol/L, P = 0.046) and borderline for TC (Δ = −2.62 vs −0.03 mmol/L, P = 0.053); per-animal values at baseline and week 5 are provided in **Supplementary Table S1**. Because baseline serum TC and LDL-C were by chance higher in the metformin group, these within-group Δ comparisons are confounded by regression to the mean and are reported descriptively only; the week 5 endpoint values are therefore the primary readout.

Together, these results establish an integrated efficacy profile encompassing improved glycemic control and pancreas-liver histopathology, forming the phenotypic basis for subsequent multi-tissue omics analyses.

### Transcriptomic landscape across three metabolic tissues and liver PC1 linkage to glycemic burden

Principal component analysis (PCA) of RNA-seq data showed dispersion across liver, skeletal muscle, and iWAT (**Figures 2A–C**; variance explained by PC1/PC2: liver 35.5%/22.5%, muscle 31.2%/21.8%, iWAT 42.1%/36.3%); in the liver, PC1 correlated inversely with 2hPG at week 5 (Spearman ρ = −0.964, exact permutation P = 0.0028; **Figure 2D**) and with FPG (ρ = −0.919, exact permutation P = 0.0063; **Figure 2E**). These correlations were supported by bootstrap 95% confidence intervals excluding zero ((−1.000, −0.698) for 2hPG and (−1.000, −0.412) for FPG) and by leave-one-out analyses (ρ ranges −1.000 to −0.943 and −0.928 to −0.870, respectively), indicating that no single animal drives them. Liver PC1 was not correlated with technical variables (sequencing depth, mapping rate, Q30: all |ρ| < 0.07, P > 0.87) or with liver RIN (ρ = −0.25, P = 0.59) (**Supplementary Figure S4**) and, notably, was not correlated with the magnitude of glycemic improvement (ΔFPG/Δ2hPG, P > 0.2), indicating that hepatic PC1 is more closely associated with endpoint disease severity than with the degree of response. We therefore used the phenotype gradient in subsequent integration analyses.

**Figure 2:**
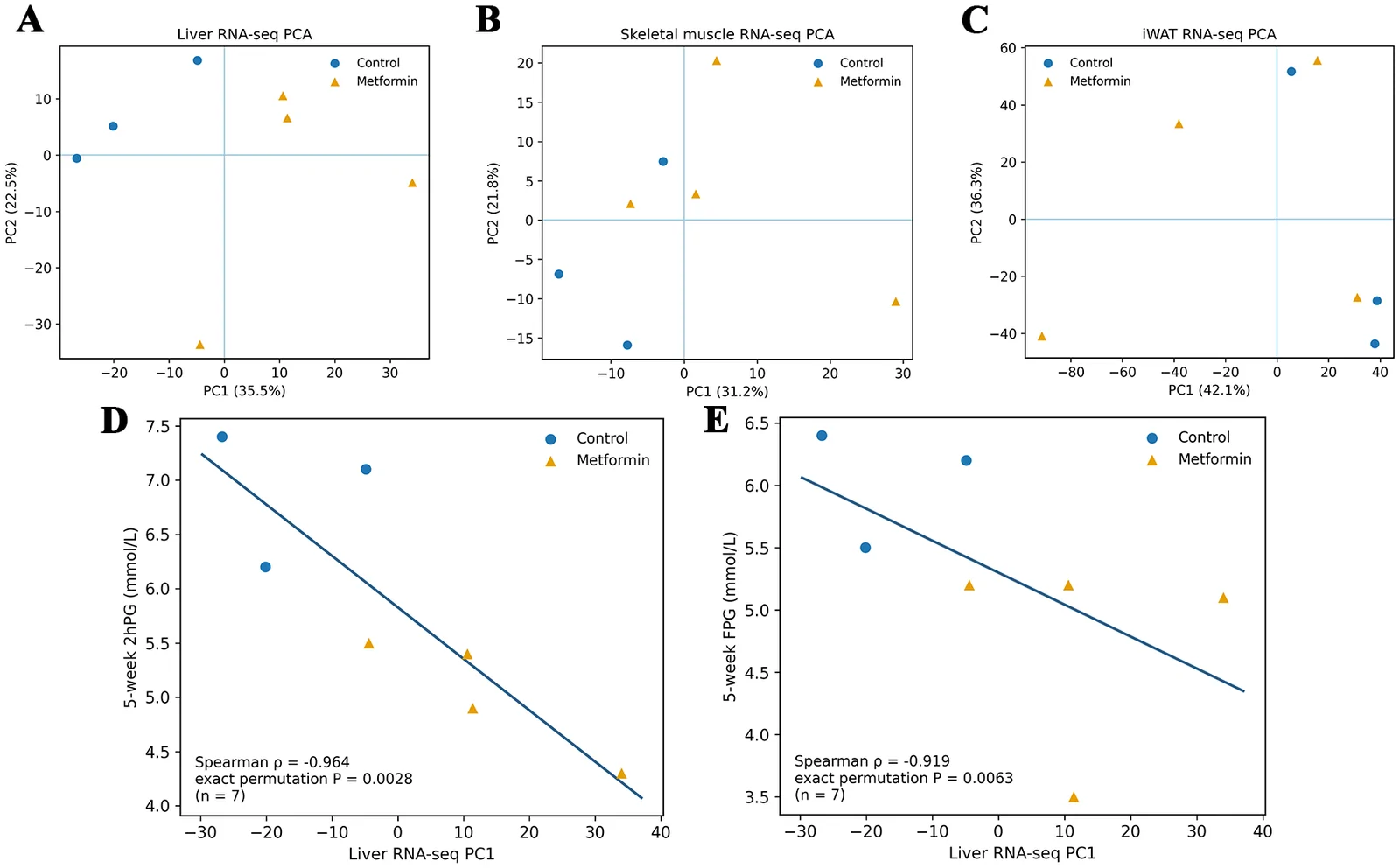
Transcriptomic landscape across metabolic tissues and the phenotype-anchored variation in the liver. **(A–C)** Principal component analysis (PCA) plots of RNA-sequencing profiles from **(A)** liver, **(B)** skeletal muscle, and **(C)** iWAT (Control, blue; Metformin, orange), illustrating the overall variance and sample dispersion within and between groups. **(D, E)** Liver transcriptomic PC1 correlates negatively with **(D)** 2hPG (Spearman ρ = −0.964, exact permutation P = 0.0028) and **(E)** FPG (Spearman ρ = −0.919, exact permutation P = 0.0063) at week 5 (n = 7), with shaded areas showing the 95% confidence interval; variance explained by PC1/PC2 was 35.5%/22.5% (liver), 31.2%/21.8% (muscle), and 42.1%/36.3% (iWAT).

### Liver: RNA-protein concordance and concordant pathway shifts

Across all matched hepatic gene products, the overall correlation between RNA and protein log2 fold changes was modest (Spearman ρ = 0.161; **Supplementary Figure S5A**); nevertheless, the 29 genes with significant changes at both levels (**Figure 3A**) significantly exceeded the chance expectation (hypergeometric P = 0.0027). Applying the stringent candidate criteria (concordant direction, |log_2_FC| ≥ 0.585 in both layers) yielded 13 hepatic genes (the 12 genes reproducible under the original imputation pipeline plus SLC25A47, which passes the nominal protein threshold only under the revised pipeline; **Supplementary Tables S3**, **S4**). These converged on the two functional modules: mitochondrial carrier family members (Slc25a47, Slc25a29, Slc25a30) and key lipid-handling genes (PLIN2, APOA5, APOC2, LCAT), alongside Lepr7 (**Figure 3A**).

**Figure 3:**
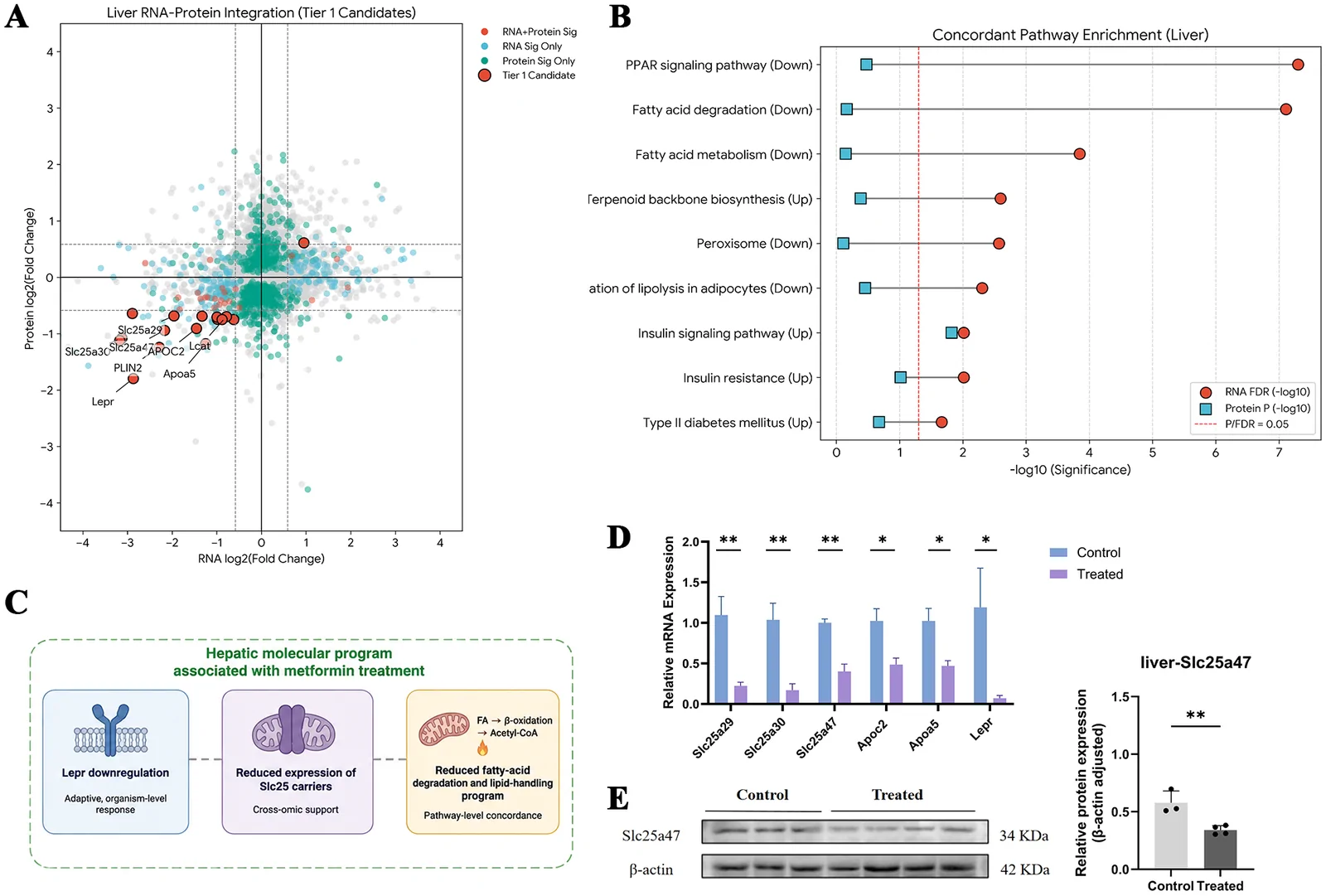
Metformin induces concordant transcriptomic and proteomic reprogramming in the liver centered on mitochondrial and lipid metabolism. **(A)** Nine-quadrant plot of hepatic RNA versus protein log2 fold changes, with exploratory cross-layer candidates (red) including Slc25a47/29/30, Plin2, Apoa5, Apoc2, and Lepr; **(B)** top concordant KEGG pathways ranked by RNA FDR (red circles) with proteomic nominal P-values (blue squares), gray lines connect the multi-omics evidence for each pathway, visualizing the directional consistency between transcriptional drivers and proteomic trends; **(C)** hepatic molecular program associated with metformin treatment, depicting the co-occurring downregulation of Lepr, reduced expression of Slc25 carriers, and a reduced fatty-acid degradation/lipid-handling program (dashed connectors denote co-occurrence within the treatment-associated program rather than a proven causal cascade; the annotation beneath each component indicates its evidence level, consistent with the adaptive nature of Lepr downregulation discussed in the text); **(D)** qRT-PCR validation of six exploratory cross-layer candidate genes (mean ± SEM, n = 3–4); Relative mRNA expression was normalized to Gapdh and presented as mean ± SEM (Control: blue bars, Treated: purple bars; n = 3–4 per group). Statistical significance was determined by two-tailed unpaired Student’s t-test: *P < 0.05, **P < 0.01. **(E)** Western blot of Slc25a47 (34 kDa) with β-actin (42 kDa) as a loading control.

Pathway enrichment showed suppression of PPAR signaling, fatty acid metabolism, and peroxisome (FDR ~10^-8^–10^-3^), whereas insulin signaling and related pathways were upregulated (FDR ~10^-2^) (**Figure 3B**). Proteomic changes, though weaker, were directionally consistent, with insulin signaling reaching nominal significance (P = 0.015). At the pathway level, the global activity correlation between omics layers did not exceed the permutation null (ρ = 0.12, P = 0.15; **Supplementary Figure S5B**), but six focused metabolic pathways (oxidative phosphorylation, thermogenesis, peroxisome, TCA cycle, fatty acid degradation, and PPAR signaling) were concordantly downregulated at both layers. Benchmarking against established metformin-responsive genes confirmed the expected directionality: Pck1 (log2FC = −1.04, FDR = 0.003), Cpt1a (−2.22), Acadm (−1.26), Cpt2 (−1.17), Acox1 (−0.83), Hadha (−0.74), and Acadl (−0.68) were all downregulated at the RNA level (all FDR < 0.05), whereas AMPKα1/α2 transcripts were unchanged, consistent with post-translational AMPK activation (**Supplementary Figure S3**). As external benchmarks, our hepatic signature was compared with two public metformin-treated transcriptomes that deliberately span very different settings: GSE190076 (Huh-7 human hepatoma cells, 20 mM metformin for 48 h *in vitro*; 16 of 20 evaluable benchmark genes directionally concordant, including all five core fatty-acid-oxidation genes) and GSE299228 (mouse liver *in vivo*, metformin 37.5 mg/kg/day incorporated into chow for 8 weeks; 12 of 17 concordant, including Cpt1a, Plin2 and Slc25a47 downregulation). Across the two datasets, 28 of 37 evaluable genes (76%) were directionally concordant (one-sided binomial P = 1.3 × 10^-3^), despite differences in species, dose, disease state and experimental design (**Supplementary Table S6**).

qRT-PCR confirmed downregulation of six exploratory cross-layer candidate genes, including Slc25a47, Slc25a29, and Plin2 (**Figure 3D**), and Western blotting confirmed reduced Slc25a47 protein in treated livers (**Figure 3E**), so the combined RNA and protein data are consistent with a metformin-associated suppression of hepatic mitochondrial carriers and lipid metabolism programs; these changes derive from the metformin-versus-vehicle comparison and are therefore treatment-associated.

Together, the gene-level candidate concordance and pathway-level agreement support a coherent model in which metformin remodels hepatic mitochondrial substrate handling and lipid trafficking programs while improving insulin-related signaling sensitivity, aligning with the histologic improvement in hepatic steatosis and the reduction in glycemic burden (**Figure 3C**).

### Skeletal muscle: cross-omics concordance and efficacy-linked module activity

Gene-level integration of skeletal muscle transcriptome and proteome data revealed limited overlap, and the overall RNA–protein log2 fold-change correlation was correspondingly weak (Spearman ρ = 0.037). Among 5, 882 matched gene products, only two genes (Qdpr and Apob) were significant at both the RNA (FDR < 0.05) and protein (nominal P < 0.05) layers, with only Apob identified as the sole exploratory cross-layer candidate (red circle) meeting strict dual-significance and effect-size criteria (|log2FC| ≥ 0.585) (**Figure 4A**; **Supplementary Figure S6B**).

**Figure 4:**
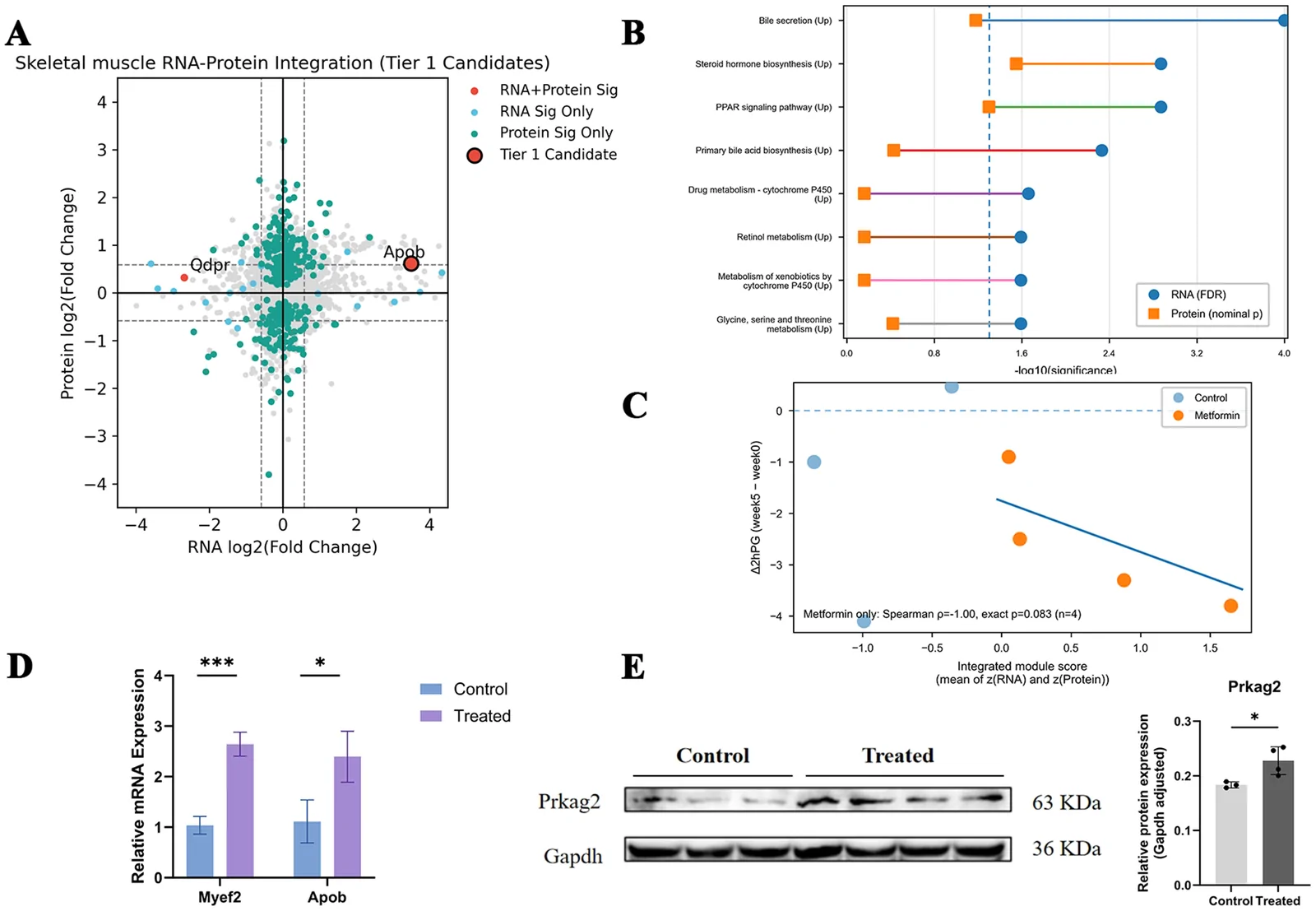
Metformin triggers coordinated activation of metabolic modules in skeletal muscle that tracks with glycemic efficacy. **(A)** The nine-quadrant plot shows limited gene-level concordance, with Apob as the sole exploratory cross-layer candidate; **(B)** KEGG pathways upregulated in RNA with consistent proteomic trends, including PPAR signaling and bile acid metabolism; **(C)** muscle PPAR module score versus Δ2hPG among treated animals (Spearman ρ = −1.00, exact permutation P = 0.083, n = 4), shown for illustrative purposes only — with n = 4, Spearman ρ takes only a limited set of discrete values, and even a perfect rank correlation cannot achieve conventional two-sided statistical significance (smallest attainable exact two-sided P = 0.083), so this exploratory correlation requires validation in larger cohorts; **(D)** qRT-PCR validation of Myef2 and Apob. Relative mRNA expression was normalized to the corresponding internal reference and presented as the mean ± SEM (Control: blue bars, Treated: orange bars; n = 3–4 per group). Statistical significance was determined by a two-tailed unpaired Student’s t-test: ***P < 0.001. **(E)** Western blot of Prkag2 (63 kDa) with Gapdh (36 kDa). Densitometric quantification of the protein bands was shown below the blot, with data presented as the mean ± SEM. Data are presented as mean ± SEM. *P < 0.05, ***P < 0.001 vs. control (Welch's t-test).

Pathway-level analysis was more informative: thirteen KEGG pathways were significantly enriched in the transcriptome with consistent proteomic trends. Upregulated pathways included PPAR signaling, bile secretion, retinol metabolism, steroid hormone biosynthesis, and cytochrome P450 drug metabolism (**Figure 4B**). Metformin suppressed the same pathways in the liver; thus, it appears to activate muscle catabolism while suppressing hepatic lipid handling.

To bridge pathway activity to efficacy, we calculated integrated module scores for each animal. Among treated animals, the PPAR signaling module correlated inversely with glycemic burden (Spearman ρ = −1.00, exact permutation P = 0.083, n = 4; **Figure 4C**) — an exploratory association, since with four animals exact P = 0.083 is the smallest achievable value. Animals with stronger muscle PPAR activation numerically had the largest glucose reductions.

qRT-PCR confirmed Myef2 and Apob upregulation in treated muscle (**Figure 4D**) and Western blotting confirmed increased Prkag2 protein, an AMPK regulatory subunit (**Figure 4E**), so, despite limited gene-level overlap, muscle responds to metformin through coordinated pathway-level metabolic activation.

### iWAT: immune/microenvironment signatures with proteomic trend support and links to inter-animal variation in glycemic response

Adipose tissue (iWAT) followed a different pattern. With 178 differentially abundant proteins but only 22 differentially expressed genes (FDR < 0.05), no exploratory cross-layer candidate, and a weak overall RNA–protein log2 fold-change correlation (Spearman ρ = 0.074), the response was largely post-transcriptional (**Figure 5A**). This transcript-versus-protein asymmetry was not attributable to inferior adipose RNA quality, as iWAT sequencing quality was comparable to that of the other tissues (Q30 96.0–96.6%).

Transcriptomic enrichment implicated immune programs, including chemokine signaling, Th17-cell differentiation, and cytokine–cytokine receptor interactions (**Figure 5B**). A rank-based proteomic trend test pointed to chemokine signaling as having the strongest cross-layer support (Trend P = 0.0467).

**Figure 5:**
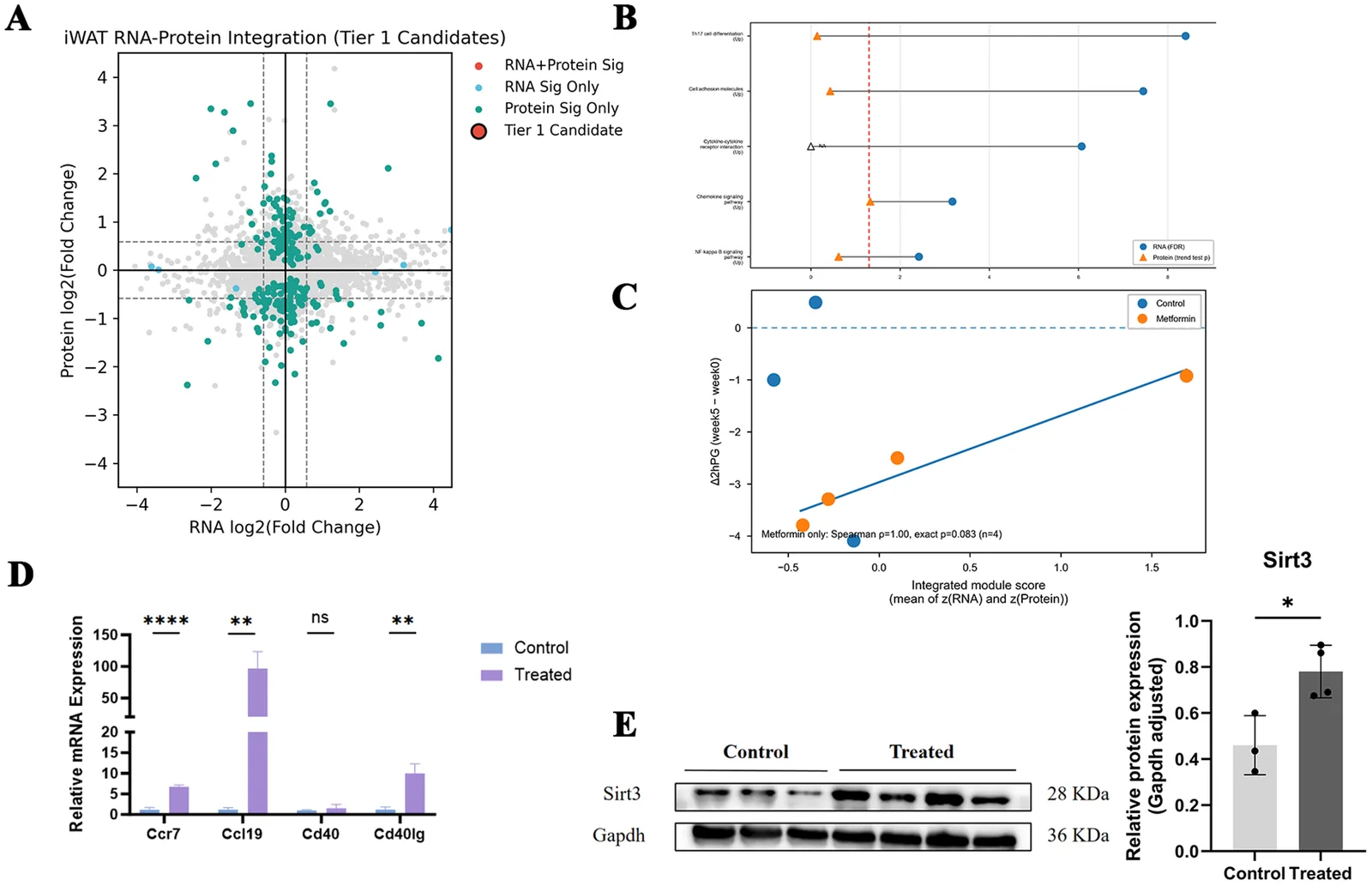
Adipose tissue immune signatures correlate with inter-animal variation in glycemic response to metformin. **(A)** The iWAT nine-quadrant plot shows broad proteomic shifts but no standard-threshold overlap with RNA; **(B)** immune pathways upregulated in RNA, with chemokine signaling showing the strongest proteomic trend support (P = 0.0467); **(C)** integrated chemokine module score versus Δ2hPG among treated animals (Spearman ρ = 1.00, n = 4; with n = 4, Spearman ρ takes only a limited set of discrete values, and even a perfect rank correlation cannot achieve conventional two-sided statistical significance — smallest attainable exact two-sided P = 0.083; exploratory); **(D)** qRT-PCR validation of Ccr7, Ccl19, Cd40, and Cd40lg; **(E)** Western blot of Sirt3 with a loading control (interpreted as a post-transcriptional mitochondrial finding rather than immunological evidence). Relative mRNA/protein expression was normalized to the corresponding internal reference and presented as the mean ± SEM. Data are presented as mean ± SEM. *P < 0.05, **P < 0.01, ****P < 0.0001 vs. control (two-sided t-test); ns, not significant.

Among treated animals, the integrated chemokine module score correlated positively with Δ2hPG (Spearman ρ = 1.00, exact permutation P = 0.083, n = 4; **Figure 5C**), an exploratory, hypothesis-generating pattern that requires confirmation. qRT-PCR confirmed upregulation of Ccr7, Ccl19, Cd40, and Cd40lg in treated iWAT (**Figure 5D**). The corresponding chemokine proteins were largely below the detection limit of the DIA workflow (**Supplementary Figure S8**). Western blot analysis confirmed increased Sirt3 protein (**Figure 5E**); because Sirt3 is a mitochondrial deacetylase without a direct role in chemokine or Th17 signaling, we interpret it as post-transcriptional regulation of mitochondrial function rather than as evidence for the immune signature. Overall, the adipose response to metformin was predominantly immunological at the transcript level, with little broad metabolic reprogramming.

### Gut microbiome profiling reveals no significant structural shifts following metformin treatment

Fecal metagenomic sequencing found no significant metformin effect on the gut microbiome. No statistically significant between-group differences in alpha-diversity indices (ACE, Chao1, Shannon, Simpson) were detected (unpaired t-test, all P > 0.05; **Supplementary Figure S1B**), and beta-diversity ordinations (PCA, NMDS) showed no treatment-related clustering (PERMANOVA, P > 0.05; **Supplementary Figure S1C, D**). Genus-level composition was comparable (**Supplementary Figure S1A**), and LEfSe identified only low-abundance, largely uncharacterized lineages enriched in the vehicle group, with no taxon of established metabolic relevance differing between groups (LDA > 2; **Supplementary Figure S1E**). In short, five weeks of metformin produced no detectable change in gut microbial ecology at this dose, duration, and sampling site in this model.

### A data-supported multi-organ model of metformin response

Across the three metabolic tissues, metformin left three recognizable fingerprints (**Figure 6**). In liver, a coherent exploratory cross-layer candidate-gene program, anchored by the downregulation of mitochondrial carriers (Slc25a family) and lipid-handling genes (Plin2, Apo series), aligns with the global transcriptomic PC1 and closely tracks the reduction in systemic glycemic burden. In skeletal muscle, coordinated activation of metabolic modules (specifically PPAR signaling and associated metabolic shifts) may relate to therapeutic efficacy, with higher module activity correlating with greater glycemic improvement among treated animals (a correlative association). In iWAT, the adipose microenvironment exhibits an upregulation of immune/chemokine signatures. Unlike muscle, higher activity of this inflammatory module associates with blunted glycemic responses among treated animals — an exploratory correlation that is compatible with, but does not prove, residual adipose inflammation acting as a brake on therapeutic benefits. Together, **Figure 6** provides a compact, evidence-anchored framework linking organ-specific molecular programs to systemic glycemic improvement and inter-animal variation in glycemic response.

**Figure 6:**
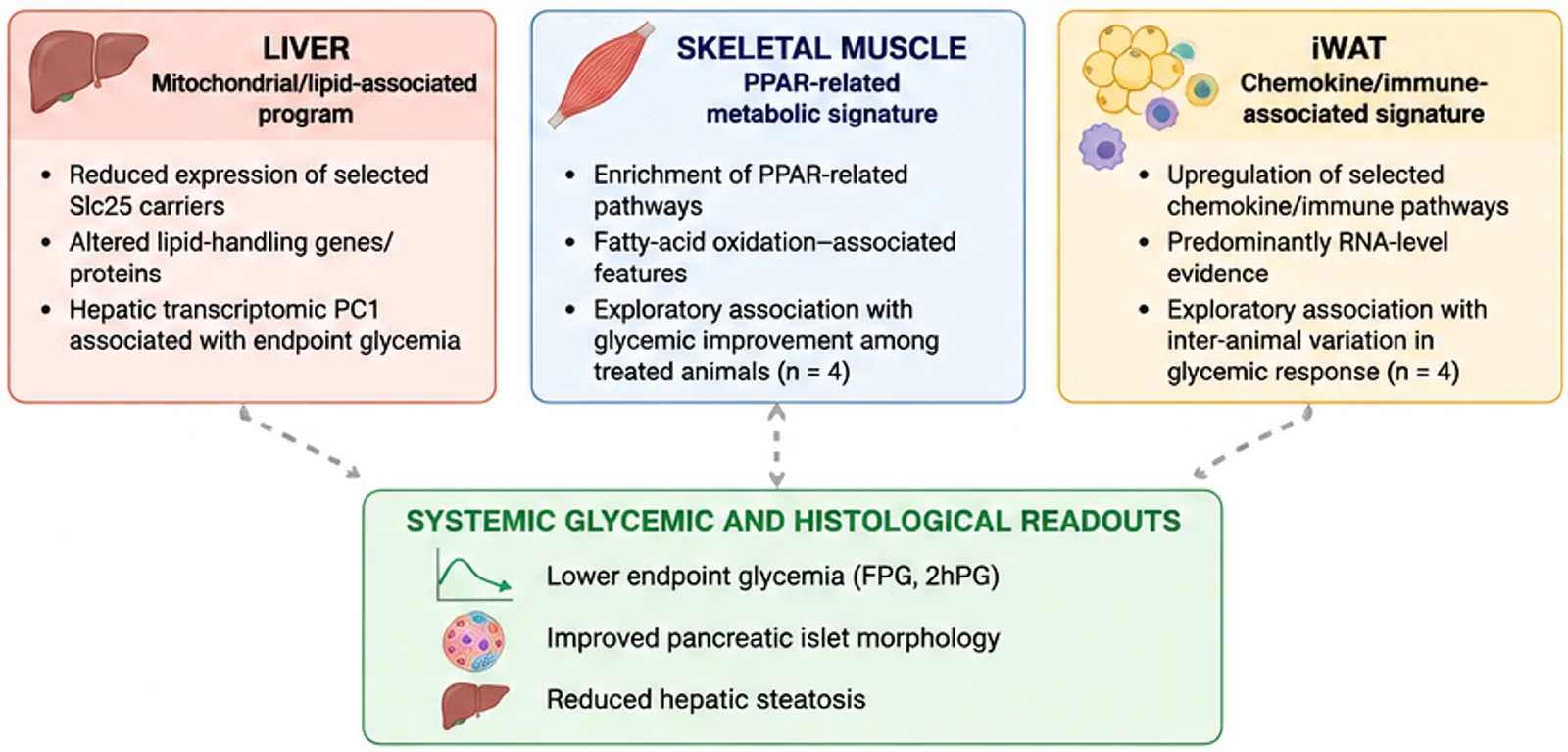
Multi-organ model of metformin efficacy and inter-animal variation in glycemic response. Schematic summarizing the organ-specific molecular programs — liver, mitochondrial/lipid-associated program; skeletal muscle, PPAR-related metabolic signature; iWAT, chemokine/immune-associated signature — together with the systemic glycemic and histological readouts (lower panel), with the evidence tier annotated for each component: concordant RNA-seq and proteomic changes (liver candidate-gene program), pathway-level transcriptomic enrichment with proteomic trend support (muscle metabolic modules; iWAT immune signatures), within-treated-group correlations (module–glycemia associations; exploratory, n = 4), and qRT-PCR/Western blot validation. Dashed bidirectional arrows denote associations between the organ programs and the systemic readouts rather than proven causal relationships.

To test multi-tissue coordination directly, we computed tissue-specific module scores for each OD animal (n = 7) at both omics layers and evaluated multi-tissue Spearman correlations (**Supplementary Figure S7A**). The hepatic candidate-gene module was tightly coupled across its RNA and protein layers (ρ = 0.86, P = 0.014). At the protein layer, the hepatic candidate-gene module was inversely correlated with the muscle PPAR-signaling module (ρ = −0.75, P = 0.052), consistent with a liver-down/muscle-up metabolic mirror that is also visible gene-by-gene along the PPAR pathway (e.g., APOA5, FABP1/7, PLIN2, PCK1, ACOX1, and EHHADH; **Supplementary Figure S7B**). The muscle PPAR module was inversely associated with the iWAT chemokine module (ρ = −0.75, P = 0.052). With n = 7 animals, the Spearman P value is determined by |ρ| alone; the two correlation pairs happened to share the same |ρ| after rounding, hence the identical P values. Given the small sample size, these multi-tissue correlations are presented as exploratory.

To systematically distinguish treatment-associated from response-associated features, we classified the 13 exploratory cross-layer candidate genes defined under the original (S0) analysis strategy (12 hepatic genes plus the muscle gene APOB) and the six tissue-level pathway modules into four categories (**Supplementary Table S3**; **Supplementary Figure S9**): (a) altered by metformin but showing no detectable correlation with the magnitude of glycemic change (LEPR, PLIN2, ECI2, and the liver protein-layer module); (b) showing exploratory correlations with glycemic change but not significantly altered by metformin (the muscle RNA- and protein-layer modules and the iWAT RNA-layer module); (c) both altered by metformin and showing exploratory correlations with the degree of glycemic change (11 features, including SLC25A29, SLC25A30, APOA5, APOC2, APOB, LCAT, and the liver RNA-layer module); and (d) showing neither (the iWAT protein-layer module). Because the four treated animals preclude conventional significance testing for within-group correlations (the smallest achievable exact P is 0.083), response-associated correlations were classified using an effect-size criterion (|ρ| ≥ 0.8) and are interpreted as exploratory observations rather than confirmatory findings.

Metformin coordinates three organ-specific programs: liver suppression of Slc25a mitochondrial carriers and lipid-handling genes aligns with reduced glycemic burden; muscle activation of PPAR and metabolic pathways tracks with greater glucose improvement; iWAT upregulation of immune/chemokine signatures correlates with residual hyperglycemia, suggesting adipose inflammation limits efficacy. Collectively, this framework proposes that metformin restores systemic homeostasis through hepatic remodeling and muscle metabolic activation, while inter-animal variation in glycemic response is shaped by the opposing interplay of adipose tissue inflammation.

## Discussion

We used tissue-resolved transcriptomic and proteomic profiling to map organ-specific responses to metformin in obese diabetic gerbils. Metformin restored systemic homeostasis through distinct molecular programs in liver, muscle, and adipose tissue. The liver showed the most coherent signature, with concordant suppression of mitochondrial carriers (Slc25a47, Slc25a29, Slc25a30) and lipid-handling genes (Plin2, Apoa5, Apoc2) at both the RNA and protein levels. Skeletal muscle responded through pathway-level activation of PPAR signaling and bile acid metabolism, whereas adipose tissue exhibited upregulation of immune and chemokine pathways. Because each signature also tracked glycemic improvement among treated animals, organ-specific programs may jointly contribute to inter-animal variation in efficacy; these associations are correlative and do not establish mediation. Multi-tissue module analysis further supported inter-organ coordination: the hepatic candidate-gene and muscle PPAR modules were inversely correlated at the protein layer (ρ = −0.75, P = 0.052), a liver-down/muscle-up metabolic mirror.

This framework links tissue-level molecular changes to individual efficacy in the same animals. Prior human multi-omics studies used single tissues or cross-sectional cohorts, so they could not distinguish causal targets from correlates (22, 23). By profiling the liver, muscle, and adipose simultaneously, we show that metformin coordinates organ-specific programs across tissues.

The hepatic response centered on Slc25a47 and related mitochondrial solute carriers. Slc25a47 encodes a mitochondrial NAD+ transporter; its suppression activates AMPKα (24). This identifies Slc25a47 as a candidate hepatic molecule associated with metformin treatment — a hypothesis that requires functional validation (e.g., testing whether manipulation of Slc25a47 alters the glycemic response to metformin). Alternatively, the reduction of Slc25a47 may represent a downstream or adaptive consequence of improved metabolic status rather than a direct pharmacological effect; our single-endpoint design cannot distinguish these possibilities. Concomitant downregulation of Plin2, Apoa5, Apoc2, and Lepr supports a lipid-clearing phenotype consistent with reduced hepatic steatosis (4–6). Coordinated suppression of Slc25a family members could slow mitochondrial substrate shuttling, cutting the fuel supply for gluconeogenesis and lipogenesis (24, 25). Because hepatic transcriptomic PC1 tracked glycemic burden, the liver functioned as the central integrator of metformin’s systemic effect. Animals with larger reductions in glycemic measures showed stronger suppression of the Slc25a47–Plin2 axis, extending the classical view of metformin as a suppressor of hepatic glucose production by linking it to mitochondrial carrier biology and lipid droplet dynamics.

In muscle, the response ran through pathway-level activation of PPAR signaling and bile acid metabolism, with minimal gene-level RNA-protein overlap. PPAR signaling rose in muscle but fell in liver, indicating tissue-specific metabolic reallocation. PPAR activation in skeletal muscle promotes fatty acid oxidation and mitochondrial biogenesis, effects that resemble exercise-induced metabolic adaptation. Apob, the sole exploratory cross-layer candidate gene in muscle, points to lipid export as a potential muscle mechanism. The sparse gene-protein overlap shows that the transcriptomic and proteomic layers diverge; pathway-level integration captures drug-induced remodeling. This finding aligns with recent work emphasizing the importance of AMPK- and PPARδ-mediated metabolic flexibility in muscle for systemic insulin sensitivity (26, 27). A study identified a muscle PPARδ signature as a predictor of exercise response in humans (27, 28). The association between muscle PPAR activity and glycemic improvement suggests, without proving, that muscle metabolic activation contributes to metformin efficacy.

Immune activation dominated the adipose response, with little metabolic reprogramming. Proteomic changes far outnumbered transcriptomic changes, so the response was largely post-transcriptional. Metformin upregulated chemokine signaling, Th17-cell differentiation, and cytokine–cytokine receptor interactions, and the integrated chemokine module score correlated positively with residual glucose. Sirt3 protein was upregulated in iWAT (log2FC = 0.82, P = 0.012) without an RNA-level change (P = 0.92), consistent with post-transcriptional regulation of mitochondrial function, and is interpreted here in the metabolic rather than the immunological context. This matches the emerging view of adipose tissue as an immunometabolic hub (29–31). Recent studies have demonstrated that specific immune cell populations in iWAT—such as CD8+ T cells and pro-inflammatory macrophages—can significantly impair metformin’s insulin-sensitizing effects (32, 33). Conversely, the same directionality is equally consistent with metformin actively recruiting anti-inflammatory or regulatory immune subsets — a documented effect of the drug — and our data cannot distinguish residual pro-inflammatory activity from adaptive immunoregulatory recruitment. Our data thus suggest a link connecting adipose immune status to inter-animal variation in glycemic response to metformin in obesity, compatible with, but not proving, a modulatory role of adipose immune activation on systemic efficacy. Although correlative, this finding provides a clear entry point for future perturbation studies, such as depletion of specific immune subsets.

The concordant downregulation of hepatic Lepr (leptin receptor) provides essential systemic context. Leptin resistance is a hallmark of this model (7), and leptin signaling mediates critical liver–adipose crosstalk (34). Lepr downregulation is unlikely to be a direct drug target; rather, it appears to be an adaptive, organism-level response to improved metabolic homeostasis (e.g., reduced adiposity and steatosis). This finding underscores that the tissue-specific molecular changes we observe occur within a dynamic framework of improved inter-organ endocrine communication, not in isolation. It reinforces the view that metformin’s overall efficacy is a product of integrated, multi-organ adaptations, not a single tissue effect.

The gut microbiome showed no significant metformin effect after five weeks of treatment, despite ongoing interest in the gut as a mediator of metformin action. This descriptive negative finding derives from single-endpoint sampling with n = 4 versus 3, without a time series or cecal-content comparison, and a permissive, exploratory LEfSe threshold; it indicates no detectable change at this dose, duration, and sampling site and should not be taken as evidence that host tissue responses dominate early glycemic improvement.

The scope of this study is deliberately hypothesis-generating, and its limitations must be explicitly acknowledged. We used nominal P values for protein-level differential testing without further multiple-testing correction, so these findings are hypothesis-generating and need independent validation. The protein layer was not FDR-controlled, creating a threshold asymmetry with the RNA layer; we therefore report candidate-gene membership under three missing-data strategies and four stringency levels (**Supplementary Table S4**; **Supplementary Figure S6C**), and the candidate set should be regarded as hypothesis-generating. Overall RNA–protein log2FC correlations across all matched gene products were weak (liver ρ = 0.161, muscle ρ = 0.037, iWAT ρ = 0.074; **Supplementary Figure S5A**); cross-layer integration is therefore restricted to the candidate core genes and the six pathway-level modules. Individual protein-level changes without an RNA counterpart (e.g., Sirt3) are presented as post-transcriptional findings, but we have no direct translation-efficiency or half-life data and do not claim global post-transcriptional concordance. The single-endpoint design cannot distinguish primary drug actions from secondary adaptations, and our proposed liver-muscle-fat model is correlative. Causal validation requires targeted perturbations, such as AAV-mediated Slc25a47 knockdown or muscle PPAR blockade. Within-group associations with ΔFPG/Δ2hPG were estimated in four treated animals and are exploratory and hypothesis-generating; they are not powered to define responder subtypes and cannot distinguish mediation from baseline differences or adaptive consequences. Exclusion of female animals and the use of a gerbil model limit generalizability, although the overlap between our findings and recent human multi-omics studies suggests that this model captures biologically relevant mechanisms. We fixed the dose and duration, and longer treatment might reveal additional microbial or adaptive changes. Fasting insulin was not measured, precluding HOMA-IR and HOMA-β assessment of whether baseline insulin sensitivity confounded the observed responses. Fortunately, the OD phenotype of this gerbil line is documented to be insulin resistant (9). Metformin was prepared from commercial tablets containing trace excipients, whereas the vehicle was double-distilled water without matched excipients. Full blinding during the dosing phase was not feasible because a single investigator both prepared the solutions and administered them; outcome assessment and data analysis were nevertheless performed blinded. Within the OD animals, skeletal-muscle RIN correlated with ΔFPG (Spearman ρ = 0.92, P = 0.004, n = 7); although all muscle samples passed vendor quality control (RIN ≥ 6.6) and no statistically significant difference in mean RIN was detected between groups, we cannot fully exclude a contribution of RNA integrity to muscle expression–phenotype covariation. Importantly, liver PC1 was not associated with liver RIN (ρ = −0.25, P = 0.59), and all liver samples showed uniformly high integrity (RIN 8.5–9.0).

Despite these limitations, organ-specific module scores are candidate features that warrant validation in independent cohorts; if validated, they could, in future larger cohorts, help stratify patients by their likely glycemic response and guide personalized metformin therapy or adjunctive treatments. Whether patients with strong adipose immune signatures might benefit from anti-inflammatory strategies similarly requires prospective validation. Future work should scale this multi-organ approach to larger cohorts, include both sexes, and test whether organ-specific modules can be validated in human biopsies or liquid biomarkers. Such translation will be essential to move from exploratory mechanisms to precision diabetes care.

## Conclusion

Metformin acts through distinct tissue-specific programs, suppressing mitochondrial carriers and lipid metabolism in the liver, switching on PPAR signaling and catabolic programs in muscle, and triggering immune pathways in fat. Each signature correlates with inter-animal variation in glycemic response; whether these tissue programs mediate metformin efficacy remains to be tested functionally. Tissue-resolved multi-omics profiling points to candidate pathway modules that warrant validation in independent cohorts before any consideration as predictive biomarkers or therapeutic targets.

## Data Availability

The datasets presented in this study can be found below: https://www.ncbi.nlm.nih.gov/, GSE341663; http://www.proteomexchange.org/, PXD081948; https://www.ncbi.nlm.nih.gov/bioproject, PRJNA1515423
