# Supplementary material for: Tissue-resolved transcriptomic and proteomic profiling reveals organ-specific molecular signatures associated with inter-animal variation in glycemic response to metformin in obese diabetic Mongolian gerbils
Source: Front Endocrinol (Lausanne). 2026 Oct 23;17:1954533. doi: 10.3389/fendo.2026.1954533 (PMC13739501; doi:10.3389/fendo.2026.1954533)
Supplement: Supplementary file 2 [file SupplementaryFile1.docx]

**Supplementary Table S1. Baseline characteristics and serum biochemistry of the study animals (OD animals)**

| **Animal** | **Group** | **Body weight D0 (g)** | **FPG week 0 (mmol/L)** | **2hPG week 0 (mmol/L)** |
| --- | --- | --- | --- | --- |
| C1 | Vehicle | 92 | 7.8 | 11.2 |
| C3 | Vehicle | 88 | 6.6 | 7.2 |
| C7 | Vehicle | 122 | 6.3 | 6.9 |
| T3 | Metformin | 118 | 5.8 | 9.2 |
| T4 | Metformin | 91 | 6.2 | 6.8 |
| T5 | Metformin | 106 | 5.9 | 8.2 |
| T9 | Metformin | 101 | 6.3 | 6.4 |
| Mean ± SEM (Vehicle) | Vehicle | 100.7 ± 10.7 | 6.90 ± 0.46 | 8.43 ± 1.39 |
| Mean ± SEM (Metformin) | Metformin | 104.0 ± 5.6 | 6.05 ± 0.12 | 7.65 ± 0.64 |
| Welch’s t-test P |  | 0.800 | 0.199 | 0.645 |

*Supplementary Table S1 (continued). Serum biochemistry at baseline (week 0) and endpoint (week 5).*

| **Animal** | **Group** | **Week** | **TC (mmol/L)** | **TG (mmol/L)** | **LDL-C (mmol/L)** | **HDL-C (mmol/L)** | **ALT (U/L)** | **AST (U/L)** |
| --- | --- | --- | --- | --- | --- | --- | --- | --- |
| T3 | Metformin | 0 | 8.00 | 1.88 | 1.88 | 4.96 | 299.6 | 604.0 |
| T3 | Metformin | 5 | 3.12 | 0.19 | 0.61 | 2.20 | 93.0 | 252.1 |
| T4 | Metformin | 0 | 5.04 | 6.36 | 1.56 | 2.32 | 129.6 | 222.0 |
| T4 | Metformin | 5 | 2.93 | 0.73 | 0.62 | 1.96 | 91.8 | 265.3 |
| T5 | Metformin | 0 | 3.48 | 0.88 | 0.80 | 1.96 | 120.0 | 597.2 |
| T5 | Metformin | 5 | 2.76 | 0.84 | 0.67 | 1.78 | 86.0 | 174.1 |
| T9 | Metformin | 0 | 5.80 | 3.90 | 1.40 | 3.10 | 214.6 | 600.6 |
| T9 | Metformin | 5 | 3.05 | 0.75 | 0.63 | 1.96 | 91.6 | 268.7 |
| C1 | Vehicle | 0 | 1.88 | 1.04 | 0.44 | 1.28 | 165.2 | 314.0 |
| C1 | Vehicle | 5 | 2.82 | 0.47 | 0.68 | 1.98 | 98.9 | 233.9 |
| C3 | Vehicle | 0 | 2.32 | 8.00 | 0.44 | 1.08 | 152.4 | 422.0 |
| C3 | Vehicle | 5 | 1.90 | 0.27 | 0.29 | 1.50 | 93.1 | 269.6 |
| C7 | Vehicle | 0 | 3.00 | 0.68 | 0.56 | 1.76 | 115.2 | 306.0 |
| C7 | Vehicle | 5 | 2.40 | 0.35 | 0.39 | 1.85 | 87.7 | 247.3 |
| Mean ± SEM (Vehicle) | Vehicle | 0 | 2.40 ± 0.33 | 3.24 ± 2.38 | 0.48 ± 0.04 | 1.37 ± 0.20 | 144.3 ± 15.0 | 347.3 ± 37.4 |
| Mean ± SEM (Metformin) | Metformin | 0 | 5.58 ± 0.94 | 3.26 ± 1.21 | 1.41 ± 0.23 | 3.08 ± 0.67 | 191.0 ± 42.0 | 506.0 ± 94.7 |
| Mean ± SEM (Vehicle) | Vehicle | 5 | 2.37 ± 0.27 | 0.36 ± 0.06 | 0.45 ± 0.12 | 1.78 ± 0.14 | 93.2 ± 3.2 | 250.3 ± 10.4 |
| Mean ± SEM (Metformin) | Metformin | 5 | 2.96 ± 0.08 | 0.63 ± 0.15 | 0.63 ± 0.01 | 1.98 ± 0.09 | 90.6 ± 1.6 | 240.1 ± 22.3 |
| Welch’s t-test P (baseline) |  | 0 | 0.037 | 0.996 | 0.024 | 0.079 | 0.358 | 0.197 |
| Welch’s t-test P (week 5) |  | 5 | 0.147 | 0.174 | 0.264 | 0.312 | 0.518 | 0.698 |
| Welch’s t-test P (Δ wk5−wk0) |  | — | 0.053 | 0.932 | 0.046 | 0.078 | 0.320 | 0.207 |

*Baseline (week 0 / day 0) values for the seven obese-diabetic (OD) animals used in the omics comparisons. Serum biochemistry (TC, TG, LDL-C, HDL-C, ALT, AST) was measured at baseline and at the endpoint (week 5). Baseline TC and LDL-C were higher in the metformin group by chance of simple randomization in this small cohort (P = 0.037 and 0.024); no other baseline variable differed significantly. At week 5 no between-group difference was significant; the within-group reduction from baseline was larger under metformin for LDL-C (P = 0.046), borderline for TC (P = 0.053), and not significant for HDL-C (P = 0.078), TG (P = 0.932), ALT (P = 0.320) or AST (P = 0.207).*

**Supplementary Table S2. RNA quality-control metrics for the 21 OD study samples (three tissues × seven animals; vendor reports, Agilent 2100 Bioanalyzer)**

| **Sample** | **Library ID** | **Tissue** | **Conc. (ng/µL)** | **28S/18S** | **RIN** | **QC result** | **Note** |
| --- | --- | --- | --- | --- | --- | --- | --- |
| Skeletal muscle C1 | FKRO240071914-1A | Skeletal muscle | 89.0 | 3.115 | 8.3 | Pass |  |
| Skeletal muscle C3 | FKRO240071916-1A | Skeletal muscle | 142.0 | 4.97 | 8.4 | Pass |  |
| Skeletal muscle C7 | FKRO240071919-1A | Skeletal muscle | 172.0 | 6.02 | 8.5 | Pass |  |
| Skeletal muscle T3 | FKRO240071921-1A | Skeletal muscle | 92.0 | 3.22 | 8.4 | Pass |  |
| Skeletal muscle T4 | FKRO240071922-1A | Skeletal muscle | 148.0 | 5.18 | 8.3 | Pass |  |
| Skeletal muscle T5 | FKRO240071923-1A | Skeletal muscle | 82.0 | 2.87 | 6.6 | Pass |  |
| Skeletal muscle T9 | FKRO240071926-1A | Skeletal muscle | 99.0 | 3.465 | 8.3 | Pass |  |
| iWAT -C1 | FKRO240071927-1A | iWAT | 28.0 | 0.98 | 7.9 | Pass |  |
| iWAT -C3 | FKRO240071929-1A | iWAT | 92.0 | 3.22 | 7.8 | Pass |  |
| iWAT -C7 | FKRO240071932-1A | iWAT | 112.0 | 3.92 | 7.1 | Pass |  |
| iWAT -T3 | FKRO240071934-1A | iWAT | 49.0 | 1.715 | 7.3 | Pass |  |
| iWAT -T4 | FKRO240071935-1A | iWAT | 157.0 | 5.495 | 7.8 | Pass |  |
| iWAT -T5 | FKRO240071936-1A | iWAT | 131.0 | 4.585 | 7.1 | Pass |  |
| iWAT -T9 | FKRO240071939-1A | iWAT | 298.0 | 10.43 | 8.2 | Pass |  |
| Liver C1 | FKRO240071953-1A | Liver | 865.0 | 30.275 | 8.5 | Pass |  |
| Liver C3 | FKRO240071955-1A | Liver | 488.0 | 17.08 | 8.7 | Pass |  |
| Liver C7 | FKRO240071958-1A | Liver | 787.0 | 27.545 | 8.8 | Pass |  |
| Liver T3 | FKRO240071960-1A | Liver | 762.0 | 26.67 | 9.0 | Pass |  |
| Liver T4 | FKRO240071961-1A | Liver | 1086.0 | 38.01 | 8.5 | Pass |  |
| Liver T5 | FKRO240071962-1A | Liver | 359.0 | 12.565 | 8.6 | Pass |  |
| Liver T9 | FKRO240071965-1A | Liver | 935.0 | 32.725 | 8.9 | Pass |  |

*RNA quality-control metrics for the 21 samples (liver, skeletal muscle and iWAT) from the seven OD animals (C1, C3, C7, T3, T4, T5, T9). All 21 samples passed vendor QC. Tissue-specific RIN ranges: liver 8.5–9.0; skeletal muscle 6.6–8.5; iWAT 7.1–8.2.*

**Supplementary Table S3. Four-class classification of exploratory cross-layer candidate genes and tissue-level pathway modules**

| **Feature** | **Layer** | **Type** | **Treatment effect (4 vs 3)** | **Treatment P** | **ρ vs ΔFPG (n = 4)** | **ρ vs Δ2hPG (n = 4)** | **Category** |
| --- | --- | --- | --- | --- | --- | --- | --- |
| LEPR | Liver_RNA | Candidate gene | -2.86955765129069 | 1.21e-12 | -0.2 | -0.6 | (a) treatment only |
| SLC25A29 | Liver_RNA | Candidate gene | -1.96213734188494 | 3.79e-12 | 0.4 | -0.8 | (c) both |
| SLC25A30 | Liver_RNA | Candidate gene | -3.12909035988481 | 7.05e-10 | 0.4 | -0.8 | (c) both |
| PLIN2 | Liver_RNA | Candidate gene | -2.28980647553808 | 5.70e-07 | 0.6 | 0.2 | (a) treatment only |
| CD274 | Liver_RNA | Candidate gene | -1.33495058349341 | 2.86e-06 | 1.0 | -0.2 | (c) both |
| LONP2 | Liver_RNA | Candidate gene | -0.622525088257957 | 1.67e-05 | 0.8 | -0.4 | (c) both |
| ECI2 | Liver_RNA | Candidate gene | -3.17447942567107 | 2.83e-05 | 0.6 | 0.2 | (a) treatment only |
| LCAT | Liver_RNA | Candidate gene | -0.785240495787186 | 3.95e-05 | 0.8 | -0.4 | (c) both |
| APOC2 | Liver_RNA | Candidate gene | -1.45605245696105 | 5.12e-05 | 0.4 | -0.8 | (c) both |
| PREX2 | Liver_RNA | Candidate gene | -0.995592413767482 | 5.99e-05 | -0.4 | -0.8 | (c) both |
| APOA5 | Liver_RNA | Candidate gene | -1.25250807956134 | 4.43e-04 | 0.4 | 0.8 | (c) both |
| THEM6 | Liver_RNA | Candidate gene | 0.950129808641438 | 8.25e-04 | 0.4 | 0.8 | (c) both |
| APOB | Muscle_RNA | Candidate gene | 3.49394058550683 | 2.44e-05 | 0.4 | -0.8 | (c) both |
| Liver RNA module | Liv_RNA | Pathway module | T 0.818 vs C -0.614 | 8.86e-04 | 0.8 | -0.4 | (c) both |
| Liver protein module | Liv_PRO | Pathway module | T 0.844 vs C -0.633 | 7.86e-04 | 0.6 | 0.2 | (a) treatment only |
| Skeletal muscle RNA module | Mus_RNA | Pathway module | T 0.058 vs C -0.044 | 7.48e-01 | 0.2 | -1.0 | (b) response only |
| Skeletal muscle protein module | Mus_PRO | Pathway module | T -0.260 vs C 0.195 | 1.94e-01 | -0.8 | -0.4 | (b) response only |
| iWAT RNA module | iWAT_RNA | Pathway module | T -0.177 vs C 0.133 | 1.68e-01 | -0.2 | 1.0 | (b) response only |
| iWAT protein module | iWAT_PRO | Pathway module | T 0.001 vs C -0.001 | 9.97e-01 | 0.6 | 0.2 | (d) neither |

*Treatment effects: DESeq2 (RNA) or Welch’s t-test (protein) for the metformin-versus-vehicle comparison within OD animals. Response associations: Spearman correlations with ΔFPG/Δ2hPG within the four metformin-treated animals. With n = 4 the smallest achievable exact P is 0.083, so response-associated classification used an effect-size criterion (|ρ| ≥ 0.8 for at least one of ΔFPG/Δ2hPG) and is exploratory. Categories follow the reviewer’s framework: (a) altered by metformin but not associated with glycemic change; (b) associated with glycemic change but not significantly altered by metformin; (c) both altered and associated; (d) neither. Module treatment effects are shown as mean z-scores (treated vs control). The 13 candidate genes classified here are those defined under the original (S0) analysis strategy (12 hepatic genes and the muscle gene APOB); SLC25A47, which passes the protein-layer threshold only under the revised S1 strategy, is listed in Supplementary Table S4 and is not part of this classification.*

**Supplementary Table S4. Candidate-gene membership under three missing-data strategies and four stringency levels**

| **Gene** | **Tissue** | **S0 original** | **S1 group-aware** | **S1 + protein BH** | **S1 + strict** |
| --- | --- | --- | --- | --- | --- |
| APOA5 | Liver | ✓ | ✓ | — | — |
| APOB | Muscle | ✓ | ✓ | — | — |
| APOC2 | Liver | ✓ | ✓ | — | — |
| CD274 | Liver | ✓ | ✓ | — | — |
| ECI2 | Liver | ✓ | ✓ | — | — |
| LCAT | Liver | ✓ | ✓ | — | — |
| LEPR | Liver | ✓ | ✓ | — | ✓ |
| LONP2 | Liver | ✓ | ✓ | — | — |
| PLIN2 | Liver | ✓ | ✓ | — | — |
| PREX2 | Liver | ✓ | ✓ | — | — |
| SLC25A29 | Liver | ✓ | ✓ | — | — |
| SLC25A30 | Liver | ✓ | ✓ | — | ✓ |
| SLC25A47 | Liver | — | ✓ | — | — |
| THEM6 | Liver | ✓ | ✓ | — | — |

*Hepatic candidate-gene membership (unique genes): 12 (S0) → 13 (S1, recommended group-aware strategy; SLC25A47 added) → 0 (S1 with protein-layer BH correction) → 2 (S1 with strict thresholds: LEPR and SLC25A30). Including the single muscle candidate APOB (retained under both S0 and S1), the across-tissue totals are 13 (S0) and 14 (S1). Key hepatic genes (SLC25A47, SLC25A29, SLC25A30, LEPR, PLIN2, APOC2) remained concordant under S1. The single muscle candidate gene (APOB) was unchanged across strategies. The protein layer is not FDR-controlled; the candidate set is hypothesis-generating.*

**Supplementary Table S5. Extended qRT-PCR primer characteristics (GC content and RefSeq templates)**

| **Gene** | **Forward (5’→3’)** | **Reverse (5’→3’)** | **F GC%** | **R GC%** | **F Tm (°C)** | **R Tm (°C)** | **Amplicon (bp)** | **RefSeq template** |
| --- | --- | --- | --- | --- | --- | --- | --- | --- |
| Slc25a47 | CGCCTCTGCTCACAGGTATTT | ATGGCCCCAGCAACAAAATC | 52.38 | 50.0 | 60.41 | 59.39 | 101 | XM_021642588.2 |
| Slc25a29 | CTCGTGGGACACCCTTTTGA | GTCTCACGCAGAAGTGTGGA | 55.0 | 55.0 | 59.89 | 59.97 | 428 | XM_021642569.2 |
| Slc25a30 | TGTGTGTGGAATCCTGTCCG | TCTGGGCTTGCATTCGGATT | 55.0 | 50.0 | 59.97 | 60.03 | 83 | XM_021635064.2 |
| Apoc2 | AGAGGAGCTGCCTGATATGG | TCCCGATTCCTGAACGTAACA | 55.0 | 47.62 | 58.65 | 59.11 | 112 | XM_021642185.2 |
| Apoa5 | AGAACTGAGCAGGGCGCAT | AGAAACTCTTCCGTGCCTGAG | 57.89 | 52.38 | 61.96 | 60.0 | 102 | XM_021639741.2 |
| Lepr | CAGACCCTGAGAGGGGTTCT | CGCACCTGCTGACAGAACTA | 60.0 | 55.0 | 60.25 | 60.04 | 192 | XM_060365981.1 |
| Myef2 | GCATTGGAATGGGACTTGGC | ATTCCTCCAAAACCCGGACC | 55.0 | 55.0 | 59.82 | 59.96 | 124 | XM_021631300.2 / XM_021631304.2 |
| Apob | GCTGAGTGTCTACTTCCACCC | TCACACGTTCCAGGAAGGATA | 57.14 | 47.62 | 60.07 | 58.47 | 148 | XM_060365608.1 |
| Cd40 | CTCTGTGGTTTCCAGCTCCG | GTTGGTTTCTTGGCCACCTTT | 60.0 | 47.62 | 60.67 | 59.52 | 122 | XM_021638646.2 |
| Cd40lg | CAGCCAAACTTCTCCCCGAT | TAAAGCGCCGACCCAATCAT | 55.0 | 50.0 | 60.04 | 60.11 | 111 | XM_021658316.2 |
| Ccl19 | CCATCACTCCCCTGAACACC | GCTAAAGGCCAGTAGGACGG | 60.0 | 60.0 | 60.04 | 60.18 | 147 | XM_060387210.1 |
| Ccr7 | TACAACGGAGTGGTTCTGGC | AGGCCAGGCTGTAGGTGATA | 55.0 | 55.0 | 59.97 | 60.03 | 112 | XM_021628597.2 |
| β-actin | AGTCGGTTGGAGCAAGCATC | GGTGAGGGGCTTCCTGTAGT | 55.0 | 60.0 | 60.67 | 60.91 | 119 | XM_021652973.2 |
| GAPDH | GACACGAGCAAGAGAGAGGC | TCAAGAGTAGGGCTCCCTGTG | 60.0 | 57.14 | 60.46 | 60.9 | 131 | XM_021636934.2 |

*All primers were designed with NCBI Primer-BLAST on Meriones unguiculatus RefSeq predicted transcripts (XM_ accessions), synthesized by Tianyi Huiyuan (Beijing, China), and used at a uniform annealing temperature of 60 °C. Specificity was verified in silico against the vendor-provided Ensembl reference library (concordant amplicons, including Ccr7, 112 bp, and Gapdh, 131 bp). Cxcl13 and Cxcr5 primer pairs were removed from revised Table 1 because these two genes were not among the qRT-PCR-validated targets.*

Supplementary Table S6. Gene-level directional benchmark of the gerbil metformin signature against two public metformin-treated transcriptomic datasets (GSE190076, Huh-7 human hepatoma cells, 20 mM metformin for 48 h in vitro; GSE299228, mouse liver after 8 weeks of metformin at 37.5 mg/kg/day incorporated into chow, beginning at 21 weeks of age).

| Gene | Gerbil log2FC (tissue) | GSE190076 Huh-7 log2FC (nominal P) | GSE299228 mouse liver log2FC (padj) | Concordant datasets (n/n evaluable) |
| --- | --- | --- | --- | --- |
| PCK1 | -1.04 (liver) | 0.00 (P=0.997) | -0.50 (P=0.888) | 1/2 |
| G6PC | — (liver) | -0.01 (P=0.423) | -0.53 (P=0.955) | — |
| CPT1A | -2.22 (liver) | 8.26 (P=0.002) | -0.10 (P=0.992) | 1/2 |
| ACADM | -1.26 (liver) | -3.37 (P=0.020) | -0.09 (P=0.964) | 2/2 |
| CPT2 | -1.17 (liver) | -0.70 (P=1.5e-04) | 0.04 (P=0.993) | 1/2 |
| ACOX1 | -0.83 (liver) | -3.61 (P=2.6e-04) | -0.02 (P=0.998) | 2/2 |
| HADHA | -0.74 (liver) | -3.88 (P=0.039) | -0.04 (P=0.993) | 2/2 |
| ACADL | -0.68 (liver) | -0.52 (P=0.044) | -0.04 (P=0.993) | 2/2 |
| SLC25A47 | -2.17 (liver) | 0.00 | -0.53 (P=0.897) | 2/2 |
| SLC25A29 | -1.96 (liver) | -0.24 (P=0.097) | — | 1/1 |
| SLC25A30 | -3.13 (liver) | -3.20 (P=0.003) | 0.44 (P=0.987) | 1/2 |
| PLIN2 | -2.29 (liver) | -13.42 (P=0.019) | -0.24 (P=0.993) | 2/2 |
| CD274 | -1.33 (liver) | -0.03 (P=0.077) | — | 1/1 |
| LONP2 | -0.62 (liver) | -1.65 (P=0.007) | -0.07 (P=0.993) | 2/2 |
| ECI2 | -3.17 (liver) | -0.61 (P=4.7e-04) | 0.07 (P=0.993) | 1/2 |
| LCAT | -0.79 (liver) | 0.46 (P=0.183) | -0.11 (P=0.980) | 1/2 |
| APOC2 | -1.46 (liver) | -0.19 (P=0.077) | 0.05 (P=0.993) | 1/2 |
| PREX2 | -1.00 (liver) | -8.17 (P=0.006) | — | 1/1 |
| APOA5 | -1.25 (liver) | -0.07 (P=0.097) | 0.08 (P=0.993) | 1/2 |
| THEM6 | 0.95 (liver) | -0.04 (P=0.130) | — | 0/1 |
| LEPR | -2.87 (liver) | -0.97 (P=0.008) | — | 1/1 |
| APOB | 3.49 (muscle) | -138.6 (P=3.0e-05) ‡ | 0.07 (P=0.993) | 1/1 |
| SIRT3 | 0.00 (liver) | -1.43 (P=0.003) | -0.07 (P=0.993) | 1/1 |
| CXCL13 | 10.90 (iWAT) | 0.00 (P=0.988) | — | — |

Notes: benchmark genes comprise the exploratory cross-layer candidate genes and canonical metformin-responsive genes. "—" = not detected or not quantified in that dataset. Concordance requires the same direction of change; genes without a treatment effect in the gerbil data (e.g., SIRT3 in liver, log2FC = 0) were not evaluable. P values for GSE190076 are nominal (uncorrected); P values for GSE299228 are Benjamini–Hochberg-adjusted. ‡ APOB in GSE190076 shows an extreme artefactual value arising from near-zero baseline counts and was excluded from concordance evaluation. Dose comparability: GSE190076 used a supra-pharmacological in vitro concentration (20 mM, 48 h) that cannot be converted to an in vivo dose and is interpreted only qualitatively; in GSE299228, metformin was given at 37.5 mg/kg/day in chow to non-diabetic, ad libitum-fed mice for 8 weeks, corresponding by body-surface-area conversion (Km = 3 for mouse versus ≈ 5 for gerbil) to ≈ 22.5 mg/kg/day in gerbils — about four-fold lower than our 100 mg/kg/day regimen. Pooled directional concordance across the two datasets: 28 of 37 evaluable genes (76%; one-sided binomial P = 1.3 × 10⁻³).

**Supplementary Figures (newly added in this revision; files provided separately)**

**Supplementary Fig. S2. Weekly glycaemic trajectories and body-weight change of OD animals (A: FPG; B: 2hPG; C: Δ body weight).**

**Supplementary Fig. S3. Benchmark of canonical metformin-responsive genes (RNA layer).**

**Supplementary Fig. S4. Omics quality control and technical variables: (A) variance explained by PC1–PC6 in the three tissues; (B) RIN overview of the 21 OD samples; (C) liver RIN versus liver PC1; (D) liver PC1 versus technical variables.**

**Supplementary Fig. S5. Cross-layer concordance: (A) RNA–protein log2FC correlations in the three tissues; (B) pathway-activity concordance versus permutation null.**

**Supplementary Fig. S6. Proteome robustness: (A) liver proteome volcano plot (strategy S1); (B) muscle proteome volcano plot (strategy S1); (C) candidate-gene membership under four stringency levels.**

**Supplementary Fig. S7. Cross-tissue analyses: (A) module-score Spearman correlation heatmap (n = 7 OD animals); (B) PPAR-pathway liver–muscle mirror.**

**Supplementary Fig. S8. Two-layer (RNA/protein) evidence for iWAT immune markers.**

**Supplementary Fig. S9. Four-class classification summary of exploratory cross-layer candidate genes and pathway modules.**
