# Supplementary material for: Tissue-resolved transcriptomic and proteomic profiling reveals organ-specific molecular signatures associated with inter-animal variation in glycemic response to metformin in obese diabetic Mongolian gerbils
Source: Front Endocrinol (Lausanne). 2026 Oct 23;17:1954533. doi: 10.3389/fendo.2026.1954533 (PMC13739501; doi:10.3389/fendo.2026.1954533)
Supplement: Supplementary file 3 [file SupplementaryFile2.docx]

**Supplementary Material**

Supplementary Fig. S1 | Gut microbiome profiles show no significant changes following metformin treatment in obese diabetic Mongolian gerbils. (A) Relative abundance of gut microbial genera at study endpoint; core dominant genera show consistent distribution between groups. (B) Alpha diversity of gut microbial communities, including species richness (ACE, upper left; Chao1, upper right) and diversity (Shannon, lower left; Simpson, lower right) indices. Data are mean ± SEM (n = 3–4 per group); no significant intergroup differences (unpaired Student's t-test, all P > 0.05). (C, D) Beta diversity visualized by PCA (C) and NMDS (D) ordinations (Bray-Curtis dissimilarity). Each point represents an individual (OD-C, blue; OD-T, red); no treatment-based clustering (PERMANOVA, P > 0.05). (E) LEfSe analysis (LDA score > 2.0): the discriminating taxa were all low-abundance, largely uncharacterized lineages enriched in the vehicle (OD-C) group, and no taxon with an established role in host glucose or drug metabolism differed between groups.

Supplementary Fig. S2 | Weekly glycaemic trajectories and body-weight change of OD animals. (A, B) Weekly fasting plasma glucose (FPG) and 2-h post-load glucose (2hPG) of the seven OD gerbils (faint lines, individual animals; bold lines with error bars, group mean ± SEM; blue circles, vehicle-OD n = 3; red squares, metformin-OD n = 4). Two-sided Welch t-tests per week: * P < 0.05, ** P < 0.01, *** P < 0.001, † P < 0.06. (C) Body-weight change from day 0 to day 35 (bars, mean ± SEM; black dots, individual animals; Welch P = 0.89).

Supplementary Fig. S3 | Benchmark of canonical metformin-responsive genes in liver RNA-seq. Hepatic log2 fold-changes (metformin vs vehicle within OD, DESeq2) for canonical metformin/AMPK-pathway and lipid-metabolism genes detected in the dataset. Red bars, FDR < 0.05; grey bars, not significant (n.s.). Numeric labels give the log2FC; * FDR < 0.05. Eight of ten detected benchmark genes were significantly down-regulated (Cpt1a −2.22, Slc25a47 −2.17, Acadm −1.26, Cpt2 −1.17, Pck1 −1.04, Acox1 −0.83, Hadha −0.74, Acadl −0.68), whereas Prkaa1/Prkaa2 were unchanged, consistent with the expected transcriptional signature of metformin.

Supplementary Fig. S4 | Quality control of the omics data and independence of the liver transcriptome from technical variables. (A) Percentage of total variance explained by PC1–PC6 computed on rlog-transformed counts (DESeq2; genes detected in ≥ 2 of 10 samples) in the three tissues of OD gerbils (vehicle n = 3, metformin n = 4 per tissue). PC1/PC2: liver 35.5%/22.5%, skeletal muscle 31.2%/21.8%, iWAT 42.1%/36.3%. Blue, liver; gold, skeletal muscle; red, iWAT. (B) RIN values (Agilent 2100 Bioanalyzer) of the 21 RNA-seq samples from the seven OD animals (three tissues each); horizontal bars, tissue means; dashed line, RIN = 7; blue, vehicle; red, metformin. (C) Liver RIN versus liver transcriptome PC1 score (OD, n = 7; Spearman ρ = −0.25, P = 0.59), indicating that RNA integrity did not drive the major axis of transcriptomic variation. (D) Liver PC1 score versus clean read count, mapping rate, Q30 and GC content for the seven OD liver samples (blue, vehicle; gold, metformin); Spearman correlations are given above each panel (clean reads ρ = −0.07, P = 0.88; mapping rate ρ = −0.04, P = 0.94; Q30 ρ = 0.04, P = 0.94; GC ρ = −0.74, P = 0.06).

Supplementary Fig. S5 | Concordance between transcript- and protein-level changes. (A) Scatter of matched RNA (DESeq2) and protein (strategy S1) log2 fold-changes (metformin vs vehicle, OD) for all quantified genes. Left, liver (Spearman ρ = 0.161, n = 7,323 genes); middle, skeletal muscle (ρ = 0.037, n = 5,882); right, iWAT (ρ = 0.074, n = 7,369). (B) Concordance between transcript- and protein-level pathway-activity scores assessed against a permutation null. Histograms: Spearman ρ distributions from 1,000 label permutations; red vertical line: observed ρ. Left, liver (observed ρ = 0.12, permutation P = 0.153); middle, skeletal muscle (ρ = −0.14, P = 0.858); right, iWAT (ρ = 0.08, P = 0.262).

Supplementary Fig. S6 | Proteome-wide changes under the recommended S1 strategy and robustness of the exploratory cross-layer candidate gene set. (A) Volcano plot of the liver proteome (OD, metformin vs vehicle), quantified with group-aware KNN imputation (≥ 50% detection per group, k = 2; strategy S1). Orange points, proteins with P < 0.05 and |log2FC| > 0.263; dashed line, P = 0.05; exploratory cross-layer candidate genes are circled and labelled. (B) Volcano plot of the skeletal-muscle proteome under the same S1 strategy; detected candidate proteins (APOB, QDPR, PRKAG2) are circled and labelled. (C) Number of candidate genes supported at the protein layer in liver (blue) and skeletal muscle (gold) under four analysis choices: S0, original global KNN imputation (k = 10); S1, group-aware filtering (≥ 50% per group) plus KNN (k = 2); S1 with protein-layer Benjamini–Hochberg correction; and a strict S1 variant (P < 0.01 and ≥ 2-fold change). The liver candidate set was stable between S0 (12) and S1 (13) in unique-gene counts (ECI2 contributes two protein records, giving record-level counts of 13 and 14); no candidate gene survived protein-layer FDR correction, consistent with the small sample size (4 vs 3).

Supplementary Fig. S7 | Multi-tissue analyses of the liver candidate-gene, muscle PPAR and iWAT chemokine modules. (A) Spearman correlations among six gene/protein modules across the three tissues (n = 7 OD animals). Cell values give Spearman ρ; * P < 0.05, † P < 0.10. Rows/columns: liver candidate genes (RNA), liver candidate genes (protein), muscle PPAR (RNA), muscle PPAR (protein), iWAT chemokine (RNA), iWAT chemokine (protein). (B) Mirror regulation of PPAR-pathway genes between liver and skeletal muscle: top 18 PPAR-pathway genes ranked by |muscle protein log2FC| (one record per gene; the entry with the largest absolute change is shown where multiple protein records exist). Bars give log2FC (metformin vs vehicle, OD) for muscle protein, muscle RNA, liver protein and liver RNA. Note the opposite directions of APOA5, FABP1/FABP7, PLIN1/PLIN2 and CPT1A between the two tissues.

Supplementary Fig. S8 | Two-layer (RNA and protein) evidence for iWAT immune markers. log2FC (metformin vs vehicle, OD) of seven immune-related markers in iWAT at the RNA (DESeq2) and protein (strategy S1) layers; values are clipped at ± 2 for display. * FDR < 0.05 (RNA) or P < 0.05 (protein); n.d., protein not detected. Cxcl13 was strongly up-regulated at the RNA level (log2FC = 10.86, FDR < 0.05); Sirt3 was significantly up-regulated at the protein level (log2FC = 0.82, P < 0.05).

Supplementary Fig. S9 | Four-class classification of exploratory cross-layer candidate genes and tissue modules by treatment effect and glycaemic response. Each feature is plotted by its treatment effect (−log10(P), metformin vs vehicle within OD; x-axis) against its within-metformin-group correlation with glycaemic improvement (Spearman ρ, n = 4; y-axis). (A) Correlation with ΔFPG; (B) correlation with Δ2hPG. Dashed vertical line, P = 0.05; dotted horizontal lines, ρ = ± 0.8. Colours: blue, (a) treatment-altered only; orange, (b) response-associated only; green, (c) both altered and response-associated; grey, neither. With n = 4, Spearman ρ takes only a limited set of discrete values, and even a perfect rank correlation cannot achieve conventional two-sided statistical significance (smallest attainable exact two-sided P = 0.083); these correlations are therefore reported as exploratory.
